# High Throughput Functional Assays of the Variant Antigen PfEMP1 Reveal a Single Domain in the 3D7 *Plasmodium falciparum* Genome that Binds ICAM1 with High Affinity and Is Targeted by Naturally Acquired Neutralizing Antibodies

**DOI:** 10.1371/journal.ppat.1000386

**Published:** 2009-04-17

**Authors:** Andrew V. Oleinikov, Emily Amos, Isaac Tyler Frye, Eddie Rossnagle, Theonest K. Mutabingwa, Michal Fried, Patrick E. Duffy

**Affiliations:** 1 Seattle Biomedical Research Institute, Seattle, Washington, United States of America; 2 National Institute for Medical Research, Dar es Salaam, Tanzania; 3 Department of Global Health, University of Washington, Seattle, Washington, United States of America; Case Western Reserve University, United States of America

## Abstract

*Plasmodium falciparum*–infected erythrocytes bind endothelial receptors to sequester in vascular beds, and binding to ICAM1 has been implicated in cerebral malaria. Binding to ICAM1 may be mediated by the variant surface antigen family PfEMP1: for example, 6 of 21 DBLβC2 domains from the IT4 strain PfEMP1 repertoire were shown to bind ICAM1, and the PfEMP1 containing these 6 domains are all classified as Group B or C type. In this study, we surveyed binding of ICAM1 to 16 DBLβC2 domains of the 3D7 strain PfEMP1 repertoire, using a high throughput Bioplex assay format. Only one DBL2βC2 domain from the Group A PfEMP1 PF11_0521 showed strong specific binding. Among these 16 domains, DBL2βC2_PF11_0521_ best preserved the residues previously identified as conserved in ICAM1-binding versus non-binding domains. Our analyses further highlighted the potential role of conserved residues within predominantly non-conserved flexible loops in adhesion, and, therefore, as targets for intervention. Our studies also suggest that the structural/functional DBLβC2 domain involved in ICAM1 binding includes about 80 amino acid residues upstream of the previously suggested DBLβC2 domain. DBL2βC2_PF11_0521_ binding to ICAM1 was inhibited by immune sera from east Africa but not by control US sera. Neutralizing antibodies were uncommon in children but common in immune adults from east Africa. Inhibition of binding was much more efficient than reversal of binding, indicating a strong interaction between DBL2βC2_PF11_0521_ and ICAM1. Our high throughput approach will significantly accelerate studies of PfEMP1 binding domains and protective antibody responses.

## Author Summary

*Plasmodium falciparum* exports the protein PfEMP1 to the surface of parasitized erythrocytes for roles in immunoevasion and adhesion. The size and structural complexity of this diverse protein family have limited earlier studies of PfEMP1 biology to low throughput and semi-quantitative approaches. We developed a high throughput quantitative assay of PfEMP1 adhesion and used it to analyze structural features of domains that bind the putative cerebral receptor ICAM1, as well as to survey the acquisition of functional antibodies in malaria-exposed children and adults. In studies of the PfEMP1 repertoire of clone 3D7 parasites, a single specific domain bound ICAM1 strongly. PfEMP1 domains that bind ICAM1 strongly have conserved features, including conserved amino acids within otherwise highly variant flexible loops of the protein. While neutralizing antibodies against the PfEMP1–ICAM1 interaction were uncommon in Tanzanian children, such antibodies were common in east African adults, possibly explaining why immune adults rarely carry ICAM1-binding parasites. This high throughput platform will significantly accelerate studies of PfEMP1 binding domains and the corresponding antibody responses involved in protective immunity.

## Introduction

The variant surface antigen *Plasmodium falciparum* erythrocyte membrane protein 1 (PfEMP1) is a virulence factor of the human malaria parasite *P. falciparum.* PfEMP1 variants are encoded by about 60 *var* genes per parasite, and have been implicated in the cytoadhesion of *P. falciparum*-infected erythrocytes (PE) to vascular endothelium [1]. PE bind numerous receptors (reviewed in [2] and [3]), including thrombospondin [4], CD36 [5], ICAM1 [6], E-selectin and VCAM-1 [7], chondroitin sulfate A (CSA) [8],[9], complement receptor 1 [10], PECAM-1 [11], heparan sulfate [12],[13], bloodgroup sugars A and B [14], and the serum proteins IgG/IgM and fibrinogen [15]. Cytoadhesion allows sequestration of PE in deep vascular beds, prevents clearance of PE in spleen, causes vascular occlusion and inflammation of different organs, and is related to cerebral malaria [16] and placental malaria [17]. PE sequestration may lead to occlusion of the microvasculature and thereby contributes to the acute pathology of severe forms of malaria [18]–[22].

Distinct domains of different *var* genes have been shown to bind specific ligands *in vitro*. For example, the CIDR1-α domain was implicated in binding CD36 [23],[24]. Different DBL1-α domains from various PfEMP1 were shown to bind the CR1 receptor on RBC [10], glycosaminoglycans on RBC, and heparan sulfate on the endothelial surface [23],[25]. The DBLβC2 domain combination binds ICAM1 [26], and DBLβ alone binds PECAM1 [23].

Specific host receptors have been implicated in specific malaria syndromes (reviewed in [27]). PE sequestration in cerebral capillaries and venules is a hallmark of cerebral malaria. ICAM1 is expressed at high levels in brains of patients with cerebral malaria, and has been implicated in this syndrome [28]. Some DBLβC2 domains of different PfEMP1 proteins bind ICAM1 [26]. In a survey of the entire repertoire of DBLβ-C2 domains (n = 25) from the IT4 line genome [29], 6 domains bound ICAM1. These studies employed a complex assay based on adhesion of ICAM1-coated beads to COS-7 cells that express PfEMP1 domains, and manual counting by microscopy. This approach is semiquantitative, time-consuming, and low throughput.

We have now developed a high throughput DBL domain-receptor binding assay and used it to study ICAM1 binding to the DBLβC2 domain repertoire of 3D7 clone parasite. Of the 16 DBLβC2 domains tested, we find that a single domain from a Group A PfEMP1 protein (PF11_0521) binds ICAM1 strongly. Our structural analyses suggest that DBL2βC2_PF11_0521_ binding to ICAM1 may be due to key conserved residues previously identified in IT4 line domains. Also, our truncation and binding analyses suggest that DBLβC2 domain extends about 80 amino acid residues upstream of its previously suggested boundary [26]. Binding-inhibition studies using our high throughput platform suggest that neutralizing antibodies may be infrequent in African children, but are common in immune African adults.

## Results

### Binding studies

In our expression system, recombinant protein levels can be monitored during immobilization/purification on immobilized anti-GFP antibodies by fluorescence, as we previously described using the multi-well plate format [30]. To immobilize DBLβC2 constructs on BioRad BioPlex beads, we used a similar scheme here (Figure S1A): anti-GFP antibody was cross-linked to beads of different fluorescence intensity (*i.e.*, different bead regions), then bead regions of distinct intensity were incubated with lysates of COS cells expressing individual domains as GFP-fusion proteins, and washed extensively for fast immobilization/purification [30]. Domain immobilization was confirmed by reactivity of beads with biotinylated anti-GFP. Signal intensity was similar for all constructs (other than mock-transfected cells, data not shown) indicating saturation of beads with recombinant domains.

**Figure 1:**
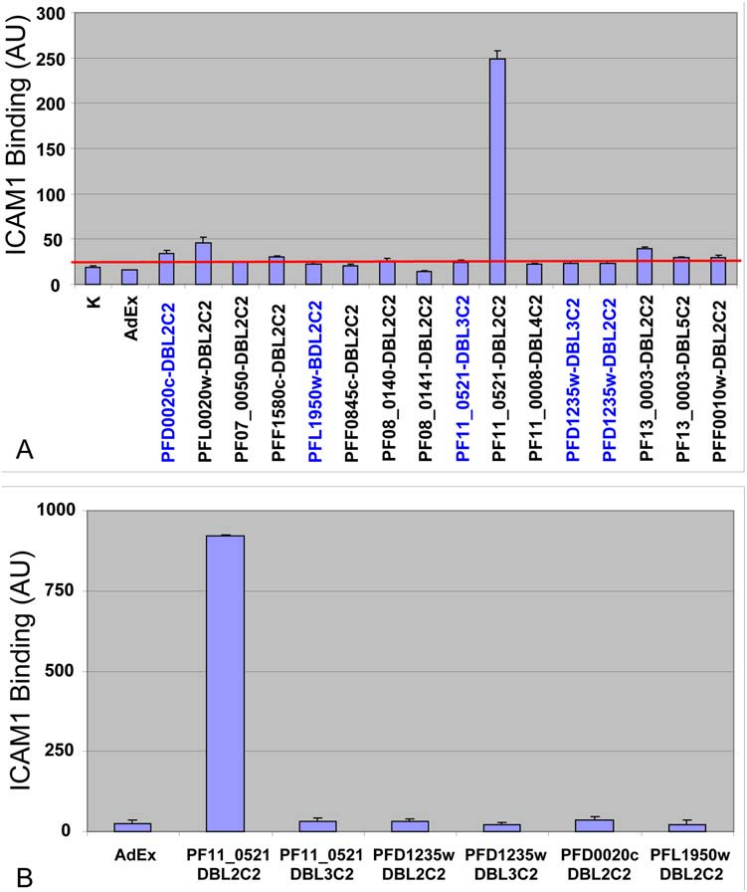
ICAM1 binds strongly only to DBL2βC2_PF11_0521_ domain from the 3D7 DBLβC2 repertoire. (A) Assays using the original panel of proteins prepared in COS-7 cells. Blue font indicates 5 constructs that were truncated to varying degrees (Figure S2) at their N-terminus in comparison with the other 11 full-length domains including the active DBL2βC2_PF11_0521_ domain. (B) Assays using full-length versions of the 5 domains that were prepared as truncated constructs in original panel of proteins. Graphs shown are representative of 3 (A) and 2 (B) independent experiments performed with different preparations of 3D7 DBLβC2 domains. Bars are averages of duplicate measurements, error bars indicate standard deviations. Results of all experiments were nearly identical. Red line indicates level of binding with the negative control beads plus 2 standard deviations. AdEx and K – negative controls in which beads were coated with lysates of pAdEx-transfected and mock-transfected COS-7 cells, respectively.

In binding assays that used this bead array (Figure S1B), only the DBL2βC2_PF11_0521_ domain among the 16 domains tested bound to ICAM1 at high levels (Figure 1). This result was reproducible in numerous assays using different preparations of recombinant domains. We also tested the DBL2βC2_PF11_0521_ domain and several other non-binding domains using the traditional multi-well plate format [30], and obtained identical results (data not shown). In addition, ICAM1-Fc binding to DBLβC2 domains was detected by anti-human IgG at a nearly identical level to detection by monoclonal antibody (mAb) RR1 (data not shown), independently confirming the above results.

**Figure 2:**
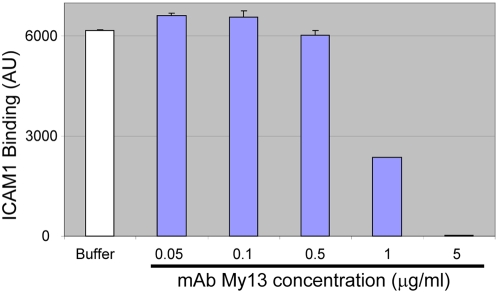
Monoclonal antibody My13 inhibits ICAM1 binding to DBL2C2_PF11_0521_. ICAM1 (1 µg/ml) was preincubated with buffer or various concentrations of mAb My13 and then tested for binding to DBL2βC2_PF11_0521_-coupled beads (average of duplicate measurements, error bars indicate standard deviations). Negative control values (signal from HisAdEx-coupled beads) were measured simultaneously in the same wells and subtracted from the signals obtained with DBL2C2_PF11_0521_ domain.

To confirm the specificity of ICAM1 binding to DBL2βC2_PF11_0521_, we tested the well-characterized mAb My13 for its ability to inhibit the interaction. According to earlier studies, My13 strongly inhibits binding of infected erythrocytes to ICAM1, but does not block binding of non-inhibitory mAb RR1 to the ICAM1 molecule [31]. Our results (Figure 2) demonstrate complete inhibition of ICAM1 binding to DBL2βC2_PF11_0521_ by an excess of My13, confirming the specificity of DBL2βC2_PF11_0521_-ICAM1 binding in our assay.

### Sequence analysis of binding domains

Using CLUSTALW 2.0.5 and subsequent manual curation, we aligned and analyzed sequences of DBLβC2 domains that bind and do not bind ICAM1. Figure S2 shows the alignment of DBLβC2 domains from both FCR3/IT and 3D7 strains, with four loops predicted to participate in ICAM1 contacts [32] indicated in boxes. We find that residues previously shown to be conserved in the ICAM1-binding DBL2βC2 domains of FCR3/IT are conserved in ICAM1-binding domain of 3D7 as well. The level of conservation among the residues in or directly adjacent to the ICAM1-binding structural loops is much higher in the binding versus non-binding DBLβC2 domains. This may indicate that these residues have an important role in structure or a direct interaction with the ligand.

Detailed analysis of the four ICAM1-binding loops revealed additional conserved residues. In loop 1, a Thr residue in the middle of the loop is conserved in every ICAM1-binding domain (and absent in 50% of non-binding domains). In loop 3, a 3-amino acid motif containing a hydrophilic residue-hydrophobic residue-hydrophilic residue, is completely conserved in binding domains. The hydrophobic residue in this motif is Ile with a single conservative exception (Val in var16), and appears to be in close contact with the ICAM1 molecule in the model [32]. This residue is absent in 37% of non-binding sequences. We speculated that preference in usage of Ile over Val may be explained by slightly larger surface of Ile in contact with the residues of ICAM1 (Figure S3A and S3B).

In previous analyses, an Ala or Leu residue was observed in position 3 of loop 4, in all but one ICAM1 binding domain (the exception being the FCR3/IT var1 DBL2βC2 domain where His is present) (Figure S2). In the 3D7 repertoire, 3 other non-binding domains carry Ala or Leu at this position, in addition to the ICAM1 binding DBL2βC2_PF11_0521_ domain. Similarly, in FCR3 strain parasites, 3 non-binding domains carry Ala or Leu at this position. However, the non-binding domains with the Ala or Leu residue in loop 4 contain multiple substitutions in other conserved regions and positions, which may explain their non-binding status.

Generally, ligand interactions involving substantial surfaces of amino acid residues are not significantly altered by substituting a single residue that participates in binding, so long as the substitution fits into the structure without clashes and does not affect structural integrity (e.g., substitutions in flexible loops) [33]. This was elegantly demonstrated by the Smith group [29], which examined amino acid substitutions and their combinations on ICAM1 binding by DBL2βC2 domains from two genes, var16 and var31 from FCR3/IT parasite. Using the model of DBL2βC2 complexed with ICAM1 [32] and the Deep View/Swiss-pdb viewer program (v.3.7), we examined the effect of replacing conserved Ala_286_ with Tyr in loop 4. The substitution does not introduce any amino acid residue clashes, and may provide an additional intra-domain hydrogen bond to R_113_ (data not shown). We infer that the loss of binding to ICAM1 is not due solely to substitutions of Ala or Leu in loop 4, but results from combinations of substitutions that involve this and other residues in the protein. We are currently testing this hypothesis using our quantitative BioPlex approach and site-specific mutagenesis. Other residues that we predict may have an effect on the domain structure or ligand binding are indicated in red in Figure S2.

### DBL2βC2_PF11_0521_ N-terminal fragment contributes to ICAM1-binding activity

Our sequence and binding analyses indicate that structural DBLβC2 domain is larger than previously suggested [26]. We propose that the domain starts about 80 aa residues upstream of the first Cys residue of A4tres DBL2βC2-ICAM1 domain. With a single exception (var6 from FCR3/IT parasite that preserves only Cys) this N-terminal region starts with a conserved Asn-Pro-Cys sequence and contains multiple conserved residues (Figure S2), independent of the type of domain located upstream of DBLβC2. With regard to downstream C2 region, our analysis demonstrates that previously described isolated DBLβ domains (e.g. DBL6β in 3D7 PFE1640, DBL5β in FCR3/IT var14, DBL3β in MC var1) are, in fact, DBLβC2 domains with degenerate C2 as well as upstream N-terminal sequences. These domains contain easily recognizable C2 features including the Y-motif and other conserved residues, as well as the upstream N-terminal fragment described above (Figure S4). A degenerate Y-motif was previously recognized in DBL6β of FCR3 VAR1CSA protein [34]. N-terminal and C2 sequences appear to diverge from the consensus sequence to similar degrees, suggesting a possible interaction between these fragments in 3-dimensional structure.

**Figure 3:**
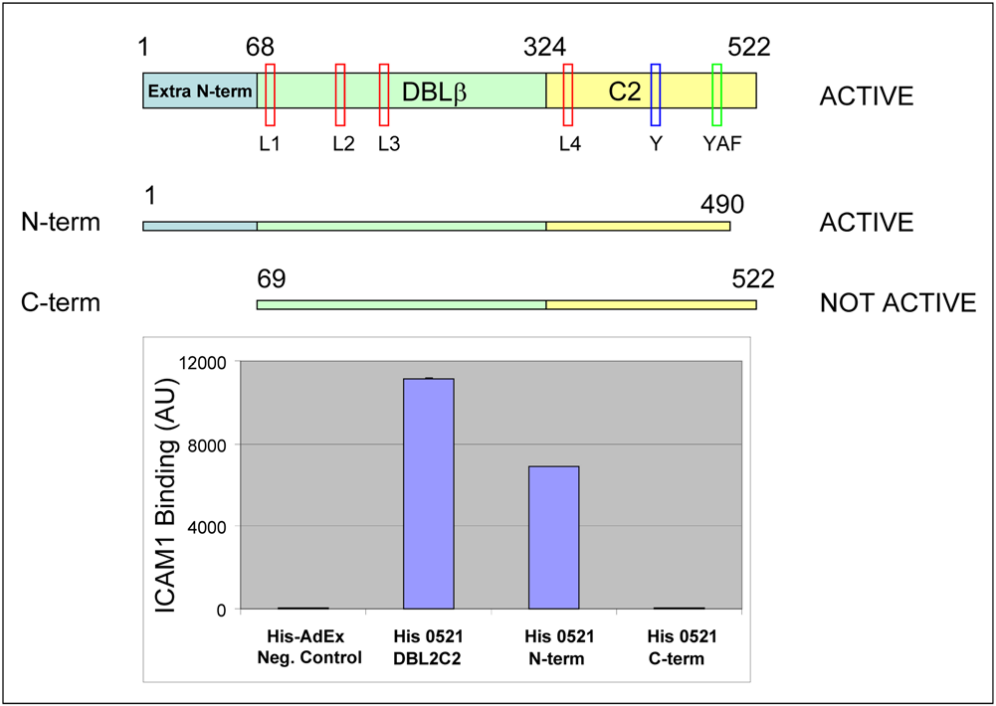
Binding activity and scheme of DBL2βC2_PF11_0521_ domain and its truncation fragments N-term and C-term. Numbers indicate amino acid residues according to Figure S2. L1 through L4 indicate positions of flexible loops shown in Figure S2. “Y” indicates position of Y-motif (Figure S4) and “YAF” indicates position of Y(A/T)F conserved motif in C2 region. Graph demonstrates ICAM1 binding activity (in arbitrary units, average of duplicate measurements) of DBL2βC2_PF11_0521_ domain and its truncated variants, N-term and C-term. Error bars indicate standard deviations. Similar results were obtained in 3 independent experiments.

We inferred that the additional N-terminal sequence contributes to the complete DBLβC2 domains, and tested its effect on ICAM1-binding activity. We re-cloned DBL2βC2_PF11_0521_ domain (amino acid residues 1–522 in Figure S2) into pHisAdEx vector as well as two truncated constructs: one (named N-term) lacked 32 amino acid residues at the C-terminus (construct ends with conserved ACNC sequence plus two residues at the C-terminus), and another one (named C-term) lacked 68 amino acid residues at the N-terminus (construct starts with conserved Asn-69 at the N-terminus and includes complete ICAM1 minimal binding domain [34]) (Figure 3). The amount of all proteins immobilized on beads was similar by reactivity with anti-GFP antibody, and all proteins had His-tag at their N-termini confirmed by reactivity with anti-His antibody (data not shown). Binding of ICAM1 (Figure 3) clearly indicate that removal of the N-terminal fragment profoundly reduces ICAM1 binding activity. Since full-length and truncated variants all demonstrated similar and strong GFP fluorescence, which is a good indicator of correct folding of the entire membrane protein [35], our results suggest an important role for the N-terminal sequence in ICAM1 binding. A similar effect on adhesion was previously observed earlier in a semi-quantitative assay of the A4tres DBL2βC2 domain with an N-terminal truncation down to the first conserved Trp (var31 Trp-106 in Figure S2). This N-terminal truncation combined with C-terminal truncation up to the end of Y-motif (var31 His-449 in Figure S2) completely abolished ICAM1 binding [34].

### Binding-inhibition and binding-reversal studies with human plasma

**Figure 4:**
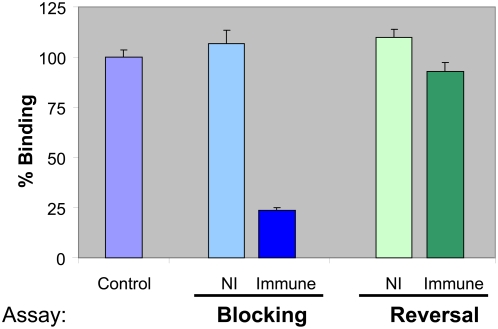
Pooled immune serum from adults living in a malaria-endemic area efficiently blocks but poorly reverses ICAM1 binding to DBL2βC2_PF11_0521_ domain. Blocking of binding: domains immobilized on BioPlex beads were preincubated with pooled US non-immune plasma (NI) or pooled immune plasma from adult patients living in malaria endemic area, then allowed to bind with ICAM1. Reversal of binding: domains immobilized on beads were allowed to bind to ICAM1, and then treated with indicated sera. Binding was normalized to the binding obtained with beads pre-incubated with buffer before reaction with ICAM1 (100%). Error bars indicate standard deviations of duplicate measurements. Similar results were obtained in 3 independent experiments.

We tested inhibition of ICAM1 binding to DBL2βC2_PF11_0521_ domain using pooled human plasma from immune adult males living in East Africa and from non-immune US adults (Figure 4). Pooled immune plasma from Africa blocked binding of ICAM1 to the DBL2βC2_PF11_0521_ domain by 78%, compared to binding in NI plasma that did not reduce binding compared to media alone. However, immune plasma was not efficient (∼15% reduced binding) in assays that measured reversal of adhesion (Figure 4), indicating a strong association between ICAM1 and the DBL2βC2_PF11_0521_ domain with low OFF rate. This result complements previous data with ICAM1-binding parasites that demonstrated less than 30% reversal of parasitized erythrocyte (PE) adhesion with immune sera [36].

**Figure 5:**
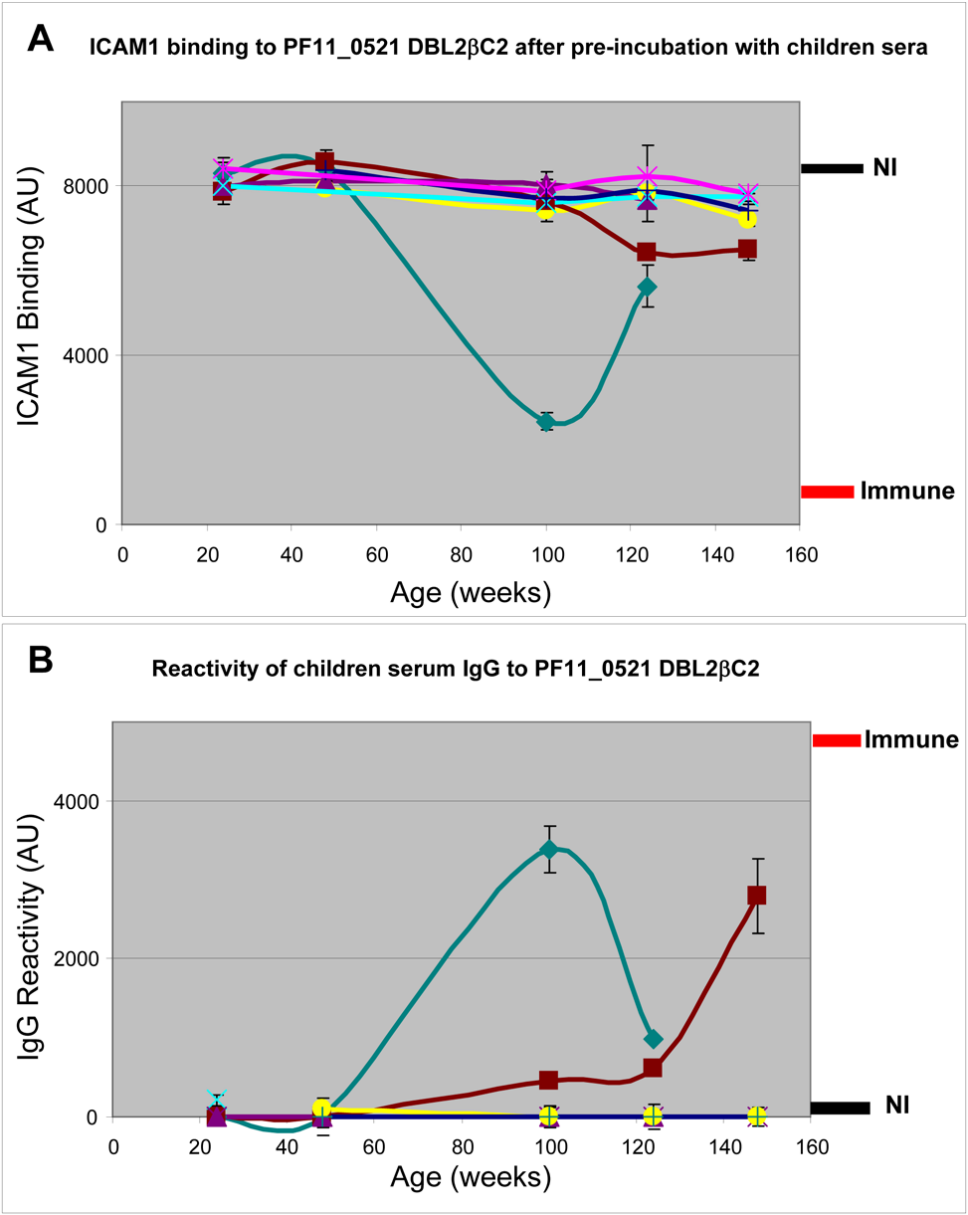
Inhibition of ICAM1 binding to DBL2βC2_PF11_0521_ domain by sera obtained from young children living in a malaria-endemic region is infrequent. Plasma samples collected from children between 24 and 148 weeks of age were used to inhibit ICAM1 binding to DBL2βC2_PF11_0521_ domain (A) and to measure the reactivity of plasma IgG to DBL2βC2_PF11_0521_ domain (B). Levels of binding after incubation with pooled plasma from non-immune (NI) or immune adults are indicated (thickness of each line corresponds to the standard deviation margin). Different colors indicate different children donating samples at sequential time points during early life. Error bars are standard deviations of duplicate measurements for each data point. Similar results were obtained in 3 independent experiments.

To study the acquisition of neutralizing antibodies against the ICAM1 binding interaction, we assayed plasma samples collected from infants and toddlers participating in longitudinal birth cohort studies in Tanzania. Plasma from 7 children that were collected at several time points between 24 and 148 weeks of age, were tested for inhibition of ICAM1 binding to DBL2βC2_PF11_0521_ domain (Figure 5A). In parallel, we tested reactivity of IgG from the same plasma to DBL2βC2_PF11_0521_ domain (Figure 5B). Inhibition of ICAM1 binding activity was uncommon, and appeared to be short-lived in at least one child. The IgG reactivity curves appear almost as mirror images of the ICAM1 binding-inhibition curves (with the exception of the 148 week time point for the brown line child, discussed below), suggesting that naturally acquired anti-DBL2βC2_PF11_0521_ domain antibodies include at least a fraction of functional antibodies as they develop in individual children.

**Figure 6:**
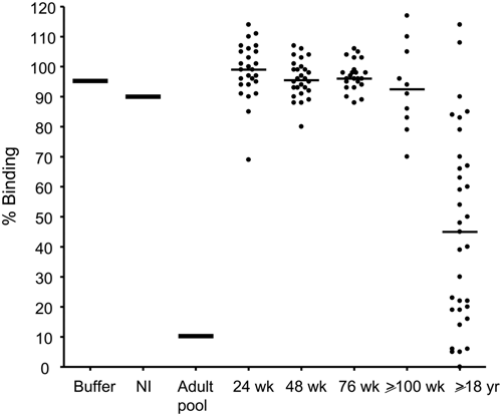
Anti-adhesion antibodies that inhibit binding of DBL2βC2_PF11_0521_ to ICAM1 accumulate with age. Individual plasma from children of various ages (24 weeks, n = 26; 48 weeks, n = 28; 76 weeks, n = 23; 100 to 148 weeks, n = 10, indicated as ≥100 wk) or adults (18 to 57 years old, n = 33) living in malaria holoendemic areas, as well as controls (buffer, pooled non-immune (NI) serum, and pooled male adult serum (Adult pool) from malaria endemic area) were used to inhibit ICAM1 binding to DBL2βC2_PF11_0521_. Horizontal bars indicate median for each group. Similar results were obtained in 2 independent experiments.

We tested levels of neutralizing antibody in additional plasma samples collected from children during the first 2 years of life, compared to plasma from adult males, all living in malaria endemic areas in East Africa (Figure 6). Plasma from most adults but only a few children contained neutralizing antibodies against the ICAM1—DBL2βC2_PF11_0521_ domain interaction, and neutralizing activity was significantly higher in plasma from adults versus children (P<0.01 when adults were compared to children 24 to 76 weeks old, and P<0.05 when compared to children 100 weeks or older, Kruskal-Wallis test). Neutralizing activity did not increase significantly in the first 2 years of life, although a trend to increasing activity was observed after 100 weeks of life. The same trend appeared in our longitudinal cohort study of 7 patients described above (Figure 5A), with a statistically significant difference in neutralizing activity between 48 and 148 weeks (P<0.05 by Kruskal Wallis test with Dunn's multiple comparison post-test).

## Discussion

PE bind endothelial receptors to sequester in vascular beds, and PE binding to ICAM1 has been implicated in cerebral malaria. In this study, we developed a functional BioPlex micro-bead protein array, and applied it to study ICAM1-binding *P. falciparum* ligands and the acquisition of neutralizing antibodies in naturally exposed individuals. Our results indicate that a single DBL2βC2_PF11_0521_ domain in the 3D7 genome binds at high levels to ICAM1, and the corresponding PfEMP1 protein is classified as Group A. Binding involves an N-terminal region that has not previously been recognized as an integral part of the DBLβC2 domain. While immune adults in East Africa commonly display neutralizing antibodies against this interaction, such antibodies are uncommon in infants and toddlers in the same region.

Immunological profiling of sera for reactivity against different antigens is a common method for assessing acquired immunity and identifying potential vaccine candidates [37]. However, relating immune responses to malaria resistance is not straightforward since exposed individuals are typically infected repeatedly throughout life, and develop diversified immune responses against multiple antigens, in many cases without comprehensible relevance to disease severity. Multiple studies have sought to relate seroreactivity with disease susceptibility in young African children [38]–[44], but no candidate antigens for a severe malaria vaccine have been identified. While seroreactivity studies are useful for defining the immunoepidemiology of existing vaccine candidate antigens [30],[45], functional assays may be essential for the discovery of novel vaccine candidates. Functional antibody responses are likely to be less diverse, to target fewer antigens, and to have a stronger association to protection from severe forms of malaria.

In this paper, we describe a high throughput approach to measure the presence and relative amount of functional antibodies in patient sera. An earlier approach, though elegant, is semi-quantitative and does not allow for high throughput studies [29]. The earlier approach was based on expression of recombinant PfEMP1 domains on the surface of mammalian cells; incubation of these mammalian cells with small resin beads chemically cross-linked to the host cell receptors; removal of unbound beads from mammalian cells attached to microscope glass by inversion and gravity sedimentation of unbound beads; and manual counting of the beads bound to the surface of mammalian cells. The approach exploited in our work is based on expression of functional antigens in mammalian cells, and rapid antigen immobilization in a directed manner on the surface of BioPlex fluorescence-coded beads. This approach allows multiplexed analyses of protein features including receptor binding activity (Figure S1) as well as seroreactivity studies in a high throughput manner.

Our studies focused on the construction of a 3D7 genome-wide array of the DBLβC2 domain, which was previously shown to bind ICAM1 in studies of other parasite lines [26],[28]. Analysis of ICAM1-binding activity in this array revealed that only the DBL2βC2_PF11_0521_ variant out of 17 domain variants, binds the receptor at high levels. Alignment of 3D7 ICAM1 binding and non-binding domains with previously identified ICAM1-binding domains from other parasite strains revealed new structural features related to the ICAM1 interaction (Figure S2), in particular, a conserved Thr residue in loop 1 and a conserved 3-amino acid motif in loop 3. This analysis further highlighted the potential role of conserved residues within predominantly non-conserved flexible loops in adhesion, and, therefore, as targets for intervention.

All DBLβC2 domains that we tested share highly conserved structural features, like helices and loops, and therefore, their general architecture should be similar. Constructs of these domains used the same boundaries and yielded recombinant protein at similar levels (according to GFP fluorescence) in a system that is well-suited for folding of transmembrane disulfide-rich proteins. Therefore, all recombinant domains have a high probability of folding similarly well. Because one of DBLβC2 domain variants clearly demonstrated binding activity, we assume that other variants that did not bind were properly folded but do not function as ICAM1 ligands. Nevertheless, since every protein is unique, false negatives can not be excluded completely without direct proof of correct folding by methods like X-ray or NMR, which are outside the scope of this study. Our findings also suggest that the structural/functional DBLβC2 domain involved in ICAM1 binding includes about 80 amino acid residues upstream of the previously suggested DBLβC2 domain [26]. This N-terminal sequence contains an alpha-helix (shown in Figure S4) predicted by several algorithms [46] in each DBLβC2 domain described here. Two other short segments associated with conserved and semi-conserved residues downstream of the alpha-helix were variously predicted to be alpha-helical or extended strands (not shown).

This high throughput assay platform can be used to profile functional antibody levels among naturally exposed children and adults. We find that antibodies that inhibit ICAM1 binding to DBL2βC2_PF11_0521_ appear sporadically in the first 2 years of life (Figure 5 and 6). Conversely, many immune adults have these antibodies in their sera. Neutralizing activity in adult plasma did not correlate with age in this group of adults (18 to 54 years old), consistent with the solid and stable protective immunity enjoyed by all adults in these communities. The slow acquisition of functional antibody may reflect that this domain variant is rare in the community, that the immature immune system of young children responds poorly to some PfEMP1, or that some other host-parasite interaction thwarts the development of functional immunity. In another study in a malaria endemic area [47], serum anti-rosetting activity against a particular lab strain (FCR3) appeared in only about 10% of children 2–5 years old, but in up to 60% of 15–16 year old adolescents, demonstrating a similar slow accumulation of functional responses. We do not know at present whether the functional response (inhibition of ICAM1 binding) is variant-specific. Future studies will clarify this question.

With regard to longevity of the immune response, an earlier study [48] found that anti-PfEMP1-like responses are short lived and variant-specific, at least in a low malaria endemicity area. We observed functional antibody to ICAM1 binding was short-lived in one child (green line in Figure 5). We are preparing to test whether this phenomenon is common using a larger set of children's plasma collected in longitudinal cohort studies. We will also examine other features of the natural immune response to malaria, such as the apparent discordance of seroreactivity and functional activity observed in some children (brown line in Figure 5). This may indicate that the amount of non-functional antibodies may increase without increasing the amount of functional antibodies, or that non-functional antibodies that increase with time may successfully compete with functional antibodies and block their activity. However, this dataset is limited and it would be premature to make definitive conclusions at present.

Our high throughput approach will now allow us to test numerous additional ICAM1 binding domains, and to determine which of these is targeted by neutralizing antibodies that also block parasite binding. With an expanded dataset, we can correlate functional antibody responses with clinical outcomes in these vulnerable populations. These future studies will also examine the concordance between this assay and the traditional binding-inhibition studies using parasitized red blood cells, including the ability to detect variant-specific versus broadly reactive functional antibodies.

## Materials and Methods

### Ethics statement

Human plasma samples used in these studies were collected from East African donors under protocols approved by relevant ethical review committees. Study participants provided written informed consent before donating samples. Ethical clearance was obtained from Institutional Review Boards of SBRI and the National Medical Research Coordinating Committee in Tanzania.

### DBLβC2 domain expression constructs

All constructs were cloned into the pAdEx vector described earlier [30]. Expression of constructs in COS-7 cells and lysate preparation were also described in [30]. All expressed constructs are GFP-fusion proteins that contain an extracellular DBLβC2 domain, a short trans-membrane region, and a cytoplasmic domain fused to green fluorescent protein (GFP). In addition, full-length and truncated forms of DBL2βC2_PF11_0521_ domain were cloned into modified vector pHisAdEx. This vector was constructed as follows: pAdEx plasmid was digested with SfiI and BamHI restriction enzymes, then the large fragment was isolated by agarose gel electrophoresis and ligated with double-stranded oligonucleotide adaptor prepared by annealing of two oligonucleotides 5′-GAT CCC TGC GTG GTG GTG GTG GTG GTG CT-3′ and 5′-ACC ACC ACC ACC ACC ACG CAG G-3′. The resulting construct was verified by sequencing. Proteins expressed from this vector are similar to proteins expressed from pAdEx vector but contain His_6_-tag at their N-termini. Various PfEMP1 domains that supported binding of ICAM1 (see above) and CD36 (data not shown) in the pAdEx expression system also supported binding in the pHisAdEx expression system. Primers used for cDNA amplification using 3D7 genomic DNA are shown in supplementary Table S1. Alignment of all DBLβC2 domains is shown in supplementary Figure S2. Five DBLβC2 domains, that were shorter at their N-termini than other 11 domains after amplification with primers indicated in Table S1, were also obtained within the same boundaries as for other domains and cloned into pAdEx and pHisAdEx for expression. New forward primers for their PCR amplifications are shown in Table S2.

### Preparation of functional DBLβC2 bead array

25 µg of anti-GFP antibody (Rockland, Gilbertsville, PA) was coupled to 200 µl of each different BioPlex bead region (Bio-Rad) (19 regions total) as described by the manufacturer, then resuspended in PBS containing 1 mg/ml BSA, 0.05% Tween-20, and 0.02% sodium azide (PBS-TBN buffer). Anti-GFP-coupled beads were incubated for 2 hours at 4°C with COS-cell lysates containing expressed domains, washed in PBS-TBN, and used in ligand binding experiments. These beads are designated as DBLβC2-coupled beads.

### DBLβC2 domain–ICAM1 binding and binding-inhibition assay

All DBLβC2-coupled beads were mixed together in quantities of ∼40–60 beads of each bead region (beads with distinct fluorescence intensity) per µl. 50 µl of bead mixtures were transferred into individual wells of HTS 96-well plates (Whatmann) that were pre-incubated with PBS-TBN for 30 minutes. Beads were washed in wells 3 times with PBS-TBN and incubated with different concentrations (20 – 0.1 µM) of ICAM1-human Fc receptor (R&D Systems, Minneapolis, MN). After 1 hour incubation at room temperature (RT) at constant rotation at 600 rpm, beads were washed in PBS-TBN and incubated in similar fashion with 1∶10 diluted biotinylated anti-ICAM1 monoclonal antibody (mAb) RR-1 (Axxora, San Diego, CA) followed by 1 hour incubation with 1∶250 diluted streptavidin-phycoerythrin (SA-PE) fluorescent molecules (Jackson ImmunoResearch, West Grove, PA). Also, in some experiments (binding only, not binding-inhibition by human serum) we used anti-human IgG coupled to phycoerythrin (1∶250 dilution, Jackson ImmunoResearch) to detect bound ICAM1-human Fc and obtained almost identical results. After a final wash, 96-well plates were transferred into the BioPlex apparatus (Bio-Rad) to quantify ICAM1 binding (measured in phycoerythrin channel) to the individual DBLβC2 domains. For negative controls, lysates prepared from mock-transfected cells, and from pAdEx vector transfected cells, were also coupled to beads. These control beads were mixed with the DBLβC2-coupled beads and assayed simultaneously. The pAdEx vector produces GFP-fusion proteins that contain an irrelevant peptide of 37 amino acids in the extracellular domain.

To confirm the specificity of ICAM1 binding to DBL2C2_PF11_0521_, ICAM1-Fc (1 µg/ml) was incubated with various concentrations of mAb My13 (Axxora) for 1 hour at room temperature and then used to bind to the mixture of His-DBL2C2_PF11_0521_ and HisAdEx (negative control) coupled beads as described above using RR1 mAb for detection.

For binding inhibition assays DBLβC2-coupled beads were pre-incubated for 1 hour at RT with various plasma samples diluted (1∶5) in PBS-TBN. The beads were then assayed in the same fashion as for the binding assay described above using RR1 mAb for detection. For binding-reversal assays, the binding assay was performed as described above, except that the beads were incubated with various plasma samples diluted (1∶5) in PBS-TBN for 1 hour at RT after the reaction with SA-PE, and then washed just prior to quantification in the BioPlex apparatus.

### Plasma samples

Human plasma samples used in these studies were collected from East African donors under protocols approved by relevant ethical review committees. Study participants provided written informed consent before donating samples, and included adult males from Kenya [49],[50] and children of different ages from Tanzania [51]. Malaria is endemic in both these regions. Plasma from 5 randomly selected non-immune donors in the US were separated from whole blood obtained from commercial sources (Valley Biomedical) and used in a pool as a negative control.

### Reactivity of human IgG with DBL2βC2_PF11_0521_ domain

DBL2βC2_PF11_0521_ domain-coupled beads were washed in wells 3 times with PBS-TBN and incubated with children plasma samples at 1∶100 dilution in PBS-TBN. After 1 hour incubation at room temperature at constant rotation at 600 rpm, beads were washed in PBS-TBN and incubated in similar fashion with 1∶250 diluted anti-human IgG coupled to phycoerythrin (Jackson ImmunoResearch) for 1 hour. Signal was measured in the BioPlex apparatus to quantify bound IgG. Signals obtained for beads coated with protein expressed by pAdEx vector were used as negative controls and were subtracted from signal obtained with DBL2βC2_PF11_0521_ domain-coupled beads. Also, pooled samples of non-immune US plasma (n = 5) and immune plasma from adults living in malaria endemic region (n = 5) were used as additional negative and positive controls, respectively.

## Supporting Information

Figure S1 Scheme of high throughput functional assays based on BioPlex technology. (A) Scheme of immobilization-purification of PfEMP1 domains on BioPlex beads. (B) Scheme of detection of receptor-binding domains and inhibition of receptor binding. Up to 100 different bead regions (colors) each coupled to 1 protein can be mixed together in 1 well for interaction with ligand. Various blocking reagents (like antibodies) can be added or pre-incubated before addition of a ligand. All beads from each well then are taken up into BioPlex machine, each bead region is classified by color (1st laser). Simultaneously, signal from detection molecule is measured by 2nd laser for each bead region (protein).
(0.07 MB DOC)

Figure S2 Alignment of 3D7 DBLβC2 domains and FCR3/IT DBLβC2 domains. FCR3/IT DBLβC2 domains are designated as var1 through var44. Numbering of residues begins with the first N-terminal residue shown in the figure for FCR3/IT domains, or with the first residue of the recombinant 3D7 domains used for assays in this study. All 3D7 domain sequences contain the minimal binding region identified previously in a semi-quantitative assay [34]. Highlighted in gray - domains that do not bind ICAM1; highlighted in dark gray - sequences that were missing from the constructs initially assayed in this work and presented in Figure 1A, they were included in constructs and tested for ICAM1 binding as described in Figure 1B; highlighted in yellow - non-binders with Ala or Leu in position 3 in flexible loop 4 (discussed in text); highlighted in green-blue - N-terminal residue and C-terminal residue of minimal var31 constructs that bind ICAM1 in semi-quantitative assay [34]. Color font: Purple - conserved and semi-conserved residues, bold font indicates predominant residue; Blue - conserved and semi-conserved residues in ICAM1 binders, bold font indicates residues only in ICAM1 binders for emphasis; Red - amino acid residue substitution with significantly different physical-chemical character that may affect structure or/and function of the domain; Green - the only exception for position 3 in loop 4 in ICAM1-binding variant; Pink - amino acid residues that differ from the annotated sequence in PlasmoDB database. Red rectangles indicate four flexible loops involved in ICAM1 binding according to the modeling studies [32].
(0.19 MB DOC)

Figure S3 Model of Ile and Val amino acid residues in “loop 3” of DBLβC2 domain interacting with ICAM1. Illustration of interaction and amino acid residue substitution were created using DBLβC2::ICAM1 complex modeled in [32] and Deep View/Swiss-pdb viewer program (v.3.7). Green - DBLβC2 domain, yellow - ICAM1 molecule. ICAM1 residues within 5 angstrom distance from Ile/Val residue of DBLβC2 domain shown in spacefill shape.
(0.31 MB DOC)

Figure S4 Alignment of two DBLβC2 domain sequences (bottom) and three DBLβ domain sequences (previously described as lacking adjacent C2 domains) with surrounding sequences (top). Color font: Orange - cysteines not present in any sequence in Figure S2, which may be involved in disulfide bonding. Other letter colors are as in Figure S2. Gray highlights indicate extra N-terminal and C2 domain sequences. Yellow highlight within C2 domain indicate “loop 4” sequence. The results demonstrate that all sequences share C2 and additional N-terminal sequence (discussed in the text) with multiple conserved residues. Green highlight above the sequence alignment indicates alpha-helix predicted for all (n = 36) DBLβC2 domains described in this work [46].
(0.06 MB DOC)

Table S1 Primers for PCR amplification of 3D7 DBLβC2 domains.
(0.05 MB DOC)

Table S2 Forward primers for PCR amplification of five 3D7 DBLβC2 domains in the same boundaries as active DBL2βC2_PF11_0521_ domain.
(0.03 MB DOC)

## References

[1] Baruch DI (1999). Adhesive receptors on malaria-parasitized red cells.. Baillieres Best Pract Res Clin Haematol.

[2] Kyes S, Horrocks P, Newbold C (2001). Antigenic variation at the infected red cell surface in malaria.. Annu Rev Microbiol.

[3] Miller LH, Baruch DI, Marsh K, Doumbo OK (2002). The pathogenic basis of malaria.. Nature.

[4] Roberts DD, Sherwood JA, Spitalnik SL, Panton LJ, Howard RJ (1985). Thrombospondin binds falciparum malaria parasitized erythrocytes and may mediate cytoadherence.. Nature.

[5] Ockenhouse CF, Tandon NN, Magowan C, Jamieson GA, Chulay JD (1989). Identification of a platelet membrane glycoprotein as a falciparum malaria sequestration receptor.. Science.

[6] Berendt AR, Simmons DL, Tansey J, Newbold CI, Marsh K (1989). Intercellular adhesion molecule-1 is an endothelial cell adhesion receptor for Plasmodium falciparum.. Nature.

[7] Ockenhouse CF, Tegoshi T, Maeno Y, Benjamin C, Ho M (1992). Human vascular endothelial cell adhesion receptors for Plasmodium falciparum-infected erythrocytes: roles for endothelial leukocyte adhesion molecule 1 and vascular cell adhesion molecule 1.. J Exp Med.

[8] Robert C, Pouvelle B, Meyer P, Muanza K, Fujioka H (1995). Chondroitin-4-sulphate (proteoglycan), a receptor for Plasmodium falciparum-infected erythrocyte adherence on brain microvascular endothelial cells.. Res Immunol.

[9] Rogerson SJ, Chaiyaroj SC, Ng K, Reeder JC, Brown GV (1995). Chondroitin sulfate A is a cell surface receptor for Plasmodium falciparum-infected erythrocytes.. J Exp Med.

[10] Rowe JA, Moulds JM, Newbold CI, Miller LH (1997). P. falciparum rosetting mediated by a parasite-variant erythrocyte membrane protein and complement-receptor 1.. Nature.

[11] Treutiger CJ, Heddini A, Fernandez V, Muller WA, Wahlgren M (1997). PECAM-1/CD31, an endothelial receptor for binding Plasmodium falciparum-infected erythrocytes.. Nat Med.

[12] Carlson J, Ekre HP, Helmby H, Gysin J, Greenwood BM (1992). Disruption of Plasmodium falciparum erythrocyte rosettes by standard heparin and heparin devoid of anticoagulant activity.. Am J Trop Med Hyg.

[13] Chen Q, Barragan A, Fernandez V, Sundstrom A, Schlichtherle M (1998). Identification of Plasmodium falciparum erythrocyte membrane protein 1 (PfEMP1) as the rosetting ligand of the malaria parasite P. falciparum.. J Exp Med.

[14] Carlson J, Wahlgren M (1992). Plasmodium falciparum erythrocyte rosetting is mediated by promiscuous lectin-like interactions.. J Exp Med.

[15] Scholander C, Treutiger CJ, Hultenby K, Wahlgren M (1996). Novel fibrillar structure confers adhesive property to malaria-infected erythrocytes.. Nat Med.

[16] Miller LH, Good MF, Milon G (1994). Malaria pathogenesis.. Science.

[17] Fried M, Duffy PE (1996). Adherence of Plasmodium falciparum to chondroitin sulfate A in the human placenta.. Science.

[18] MacPherson GG, Warrell MJ, White NJ, Looareesuwan S, Warrell DA (1985). Human cerebral malaria. A quantitative ultrastructural analysis of parasitized erythrocyte sequestration.. Am J Pathol.

[19] Carlson J, Helmby H, Hill AV, Brewster D, Greenwood BM (1990). Human cerebral malaria: association with erythrocyte rosetting and lack of anti-rosetting antibodies.. Lancet.

[20] Rowe A, Obeiro J, Newbold CI, Marsh K (1995). Plasmodium falciparum rosetting is associated with malaria severity in Kenya.. Infect Immun.

[21] Heddini A, Pettersson F, Kai O, Shafi J, Obiero J (2001). Fresh isolates from children with severe Plasmodium falciparum malaria bind to multiple receptors.. Infect Immun.

[22] Roberts DJ, Pain A, Kai O, Kortok M, Marsh K (2000). Autoagglutination of malaria-infected red blood cells and malaria severity.. Lancet.

[23] Chen Q, Heddini A, Barragan A, Fernandez V, Pearce SF (2000). The semiconserved head structure of Plasmodium falciparum erythrocyte membrane protein 1 mediates binding to multiple independent host receptors.. J Exp Med.

[24] Robinson BA, Welch TL, Smith JD (2003). Widespread functional specialization of Plasmodium falciparum erythrocyte membrane protein 1 family members to bind CD36 analysed across a parasite genome.. Mol Microbiol.

[25] Vogt AM, Barragan A, Chen Q, Kironde F, Spillmann D (2003). Heparan sulfate on endothelial cells mediates the binding of Plasmodium falciparum-infected erythrocytes via the DBL1alpha domain of PfEMP1.. Blood.

[26] Smith JD, Craig AG, Kriek N, Hudson-Taylor D, Kyes S (2000). Identification of a Plasmodium falciparum intercellular adhesion molecule-1 binding domain: a parasite adhesion trait implicated in cerebral malaria.. Proc Natl Acad Sci U S A.

[27] Flick K, Chen Q (2004). var genes, PfEMP1 and the human host.. Mol Biochem Parasitol.

[28] Turner GD, Morrison H, Jones M, Davis TM, Looareesuwan S (1994). An immunohistochemical study of the pathology of fatal malaria. Evidence for widespread endothelial activation and a potential role for intercellular adhesion molecule-1 in cerebral sequestration.. Am J Pathol.

[29] Howell DP, Levin EA, Springer AL, Kraemer SM, Phippard DJ (2008). Mapping a common interaction site used by Plasmodium falciparum Duffy binding-like domains to bind diverse host receptors.. Mol Microbiol.

[30] Oleinikov AV, Rossnagle E, Francis S, Mutabingwa TK, Fried M (2007). Effects of sex, parity, and sequence variation on seroreactivity to candidate pregnancy malaria vaccine antigens.. J Infect Dis.

[31] Berendt AR, McDowall A, Craig AG, Bates PA, Sternberg MJ (1992). The binding site on ICAM-1 for Plasmodium falciparum-infected erythrocytes overlaps, but is distinct from, the LFA-1-binding site.. Cell.

[32] Bertonati C, Tramontano A (2007). A model of the complex between the PfEMP1 malaria protein and the human ICAM-1 receptor.. Proteins.

[33] Weber G, Weber G (1992). Protein interactions;.

[34] Springer AL, Smith LM, Mackay DQ, Nelson SO, Smith JD (2004). Functional interdependence of the DBLbeta domain and c2 region for binding of the Plasmodium falciparum variant antigen to ICAM-1.. Mol Biochem Parasitol.

[35] Drew DE, von Heijne G, Nordlund P, de Gier JW (2001). Green fluorescent protein as an indicator to monitor membrane protein overexpression in Escherichia coli.. FEBS Lett.

[36] Gardner JP, Pinches RA, Roberts DJ, Newbold CI (1996). Variant antigens and endothelial receptor adhesion in Plasmodium falciparum.. Proc Natl Acad Sci USA.

[37] Bacarese-Hamilton T, Bistoni F, Crisanti A (2002). Protein microarrays: from serodiagnosis to whole proteome scale analysis of the immune response against pathogenic microorganisms.. Biotechniques.

[38] Magistrado PA, Lusingu J, Vestergaard LS, Lemnge M, Lavstsen T (2007). Immunoglobulin G antibody reactivity to a group A Plasmodium falciparum erythrocyte membrane protein 1 and protection from P. falciparum malaria.. Infect Immun.

[39] Joergensen L, Vestergaard LS, Turner L, Magistrado P, Lusingu JP (2007). 3D7-Derived Plasmodium falciparum erythrocyte membrane protein 1 is a frequent target of naturally acquired antibodies recognizing protein domains in a particular pattern independent of malaria transmission intensity.. J Immunol.

[40] Osier FH, Fegan G, Polley SD, Murungi L, Verra F (2008). Breadth and magnitude of antibody responses to multiple Plasmodium falciparum merozoite antigens are associated with protection from clinical malaria.. Infect Immun.

[41] Dobano C, Rogerson SJ, Mackinnon MJ, Cavanagh DR, Taylor TE (2008). Differential antibody responses to Plasmodium falciparum merozoite proteins in Malawian children with severe malaria.. J Infect Dis.

[42] Nasr A, Iriemenam NC, Troye-Blomberg M, Giha HA, Balogun HA (2007). Fc gamma receptor IIa (CD32) polymorphism and antibody responses to asexual blood-stage antigens of Plasmodium falciparum malaria in Sudanese patients.. Scand J Immunol.

[43] Duarte J, Deshpande P, Guiyedi V, Mecheri S, Fesel C (2007). Total and functional parasite specific IgE responses in Plasmodium falciparum-infected patients exhibiting different clinical status.. Malar J.

[44] Schreiber N, Brattig N, Evans J, Tsiri A, Horstmann RD (2006). Cerebral malaria is associated with IgG2 and IgG4 antibody responses to recombinant Plasmodium falciparum RIFIN antigen.. Microbes Infect.

[45] Salanti A, Dahlback M, Turner L, Nielsen MA, Barfod L (2004). Evidence for the involvement of VAR2CSA in pregnancy-associated malaria.. J Exp Med.

[46] Combet C, Blanchet C, Geourjon C, Deleage G (2000). NPS@: network protein sequence analysis.. Trends Biochem Sci.

[47] Barragan A, Kremsner PG, Weiss W, Wahlgren M, Carlson J (1998). Age-related buildup of humoral immunity against epitopes for rosette formation and agglutination in African areas of malaria endemicity.. Infect Immun.

[48] Giha HA, Staalsoe T, Dodoo D, Elhassan IM, Roper C (1999). Nine-year longitudinal study of antibodies to variant antigens on the surface of Plasmodium falciparum-infected erythrocytes.. Infect Immun.

[49] Kurtis JD, Lanar DE, Opollo M, Duffy PE (1999). Interleukin-10 responses to liver-stage antigen 1 predict human resistance to Plasmodium falciparum.. Infect Immun.

[50] Fried M, Muga RO, Misore AO, Duffy PE (1998). Malaria elicits type 1 cytokines in the human placenta: IFN-gamma and TNF-alpha associated with pregnancy outcomes.. J Immunol.

[51] Mutabingwa TK, Bolla MC, Li JL, Domingo GJ, Li X (2005). Maternal Malaria and Gravidity Interact to Modify Infant Susceptibility to Malaria.. PLoS Med.

