# Supplementary material for: High Throughput Functional Assays of the Variant Antigen PfEMP1 Reveal a Single Domain in the 3D7 Plasmodium falciparum Genome that Binds ICAM1 with High Affinity and Is Targeted by Naturally Acquired Neutralizing Antibodies
Source: PLoS Pathog. 2009 Apr 17;5(4):e1000386. doi: 10.1371/journal.ppat.1000386 (PMC2663049; doi:10.1371/journal.ppat.1000386)
Supplement: Figure S2 — Alignment of 3D7 DBLβC2 domains and FCR3/IT DBLβC2 domains. FCR3/IT DBLβC2 domains are designated as var1 through var44. Numbering of residues begins with the first N-terminal residue shown in the figure for FCR3/IT domains, or with the first residue of the recombinant 3D7 domains used for assays in this study. All 3D7 domain sequences contain the minimal binding region identified previously in a semi-quantitative assay [34]. Highlighted in gray - domains that do not bind ICAM1; highlighted in dark gray - sequences that were missing from the constructs initially assayed in this work and presented in Figure 1A, they were included in constructs and tested for ICAM1 binding as described in Figure 1B; highlighted in yellow - non-binders with Ala or Leu in position 3 in flexible loop 4 (discussed in text); highlighted in green-blue - N-terminal residue and C-terminal residue of minimal var31 constructs that bind ICAM1 in semi-quantitative assay [34]. Color font: Purple - conserved and semi-conserved residues, bold font indicates predominant residue; Blue - conserved and semi-conserved residues in ICAM1 binders, bold font indicates residues only in ICAM1 binders for emphasis; Red - amino acid residue substitution with significantly different physical-chemical character that may affect structure or/and function of the domain; Green - the only exception for position 3 in loop 4 in ICAM1-binding variant; Pink - amino acid residues that differ from the annotated sequence in PlasmoDB database. Red rectangles indicate four flexible loops involved in ICAM1 binding according to the modeling studies [32]. (0.19 MB DOC) [file ppat.1000386.s002.doc]

**CLUSTAL 2.0.5 multiple sequence alignment for DBLβC2 domains**

PF11_0521_D2 ------**NPC**AKPHGKKLAT-----------**V**KQ**IA**QYYK**R**K**A**YIQ**L**NE**R**----------- 32

var16 ------**NPC**AEPNGSNTKHRAI--------**A**HN**VA**YHIQ**K**D**A**HAE**A**SK**R**----------- 35

var13 ------**NPC**VVGGQADTIYPAI--------**A**NQ**MA**HQMH**E**D**A**QTE**A**SK**R**----------- 35

Var14 ------**NPC**SGTRHRAM-------------**V**KN**VA**ADMY**R**A**A**RQQ**L**RN**R**----------- 30

Var31 (A4tres) ------**NPC**GKTDGTTVR------------**A**KQ**IA**KKFQ**R**D**A**KTQ**M**KNNT**R**NDGTGR--- 39

var27 ------**NPC**SGESGNKRYPVL---------**A**NK**VA**YQMH**H**K**A**KTQ**L**AS**R**----------- 34

Var1 ------**NPC**SGDPGGNTTHRSI--------**V**KT**VA**REMH**R**K**A**HQE**A**KG**R**----------- 35

var12 ------**NPC**AKPSGSAHRAL----------**V**NK**VA**SNMH**H**K**K**KRQ**L**VNR----------- 33

var17 ------**NPC**SGE-SGKKLYPVL--------**A**GK**MA**YQMH**V**A**A**KTG**L**GGNRN--------- 37

var44 ------**NPC**SGDKTGDQRYEAV--------**A**QT**VA**KILQ**Q**K**A**HTD**M**LQRSGKNGDS---- 42

Var2 ------**NPC**VNSRDQKVGNVKS--------**V**RD**VA**EEMQ**K**E**V**KKG**M**LERSVKEGDK---- 42

Var7 ------**NPC**INNTTTTSGGNNKHAT-----**V**KQ**IA**QYYK**R**Q**A**YSE**A**NNRSD--------- 40

var8 ------**NPC**VTVGDDTRGSSGKIKS-----**V**RQ**VA**KEMQ**R**EGSVRHDGDI---------- 39

var11 ------**NPC**ANPSGIKHPVL----------**A**TK**VA**HQMQ**Q**K**A**HQK**M**IENSVKNSEIGKGH 44

Var19 ------**NPC**IKGTRKRTPKRTGGASNNLVS**V**KH**IA**EIMQ**Q**D**A**REQ**L**EKR----------- 43

var15 ------**NPC**YGNNTYDAL------------**A**GK**VA**QKLQ**Q**E**A**KEQ**L**DRND---------- 32

Var20 ------**NPC**GKNPSASNNLVR---------**V**KR**LA**EMMQ**R**Y**A**RKQ**L**EKR----------- 34

var6 ------GT**C**KRTGGASNNLVS---------**V**KH**IA**EIMQ**Q**D**A**REQ**L**EKR----------- 34

var10 ------**NPC**YSDTTTEYAVL----------**A**GK**VA**QKFQ**G**E**V**RAK**M**LERSRKNGET---- 40

PF08_0141_D2 ---PTP**NPC**VNGGDDTSGAQITS-------**V**TE**IA**EGMH**M**G**A**QKQ**M**LERSGDKSGK---- 46

PF11_0008_D4 ------**NPC**GDKSATNVVN-----------**V**TE**VA**KEMH**E**E**A**HKD**M**LERSVKKVES---- 38

PF11_0521_D3 ------**NPC**VRKDQSGTHIVS---------**V**ED**VA**QGMQ**R**E**T**HDR**V**TKV----------- 26

PF13_0003_D2 --PRAH**NPC**VDKNDSQPTKT----------**V**SY**IA**RQMH**R**R**A**KAQ**M**TKNSVVDGDN---- 44

PF13_0003_D5 ----PP**NPC**GDKDATNVVG-----------**V**EV**LA**KEMQ**E**A**A**HKS**M**LSRSAVDSGKG--- 42

PFD0020c_D2 ------**NPC**GTNNNGGKLVR----------**V**KR**LA**EMMQ**R**R**A**RKQ**L**EKR----------- 33

PFD1235w_D3 ------**NPC**AETGGVHTIKT----------**V**TD**VA**KILQ**G**E**A**NET**M**LKN----------- 33

PFD1235w_D2 ------**NPC**AKPPGSKPTKS----------**V**KQ**LA**EHMQ**Q**K**A**QKL**L**GTR**-**---------- 33

PF08_0140_D2 ------**NPC**GKNNNGGKLVR----------**V**KR**PA**ELKQ**Q**K**A**HIQ**L**EKR----------- 33

PFF0010w_D2 EEDENCPKTKI**NPC**IKRTRIPTRGASNNLVS----**V**KH**IA**ELMQ**R**S**A**RKQ**L**EAG----------- 50

PFF1580c_D2 -----S**NPC**ATPSGSYPSL-----------**A**NK**VA**QLMH**H**K**A**KTQ**L**AIR----------- 33

PFL0020w_D2 ------**NPC**SAQPGGRYTVR----------**V**KD**IA**KQMH**R**R**A**KTQ**M**RNNSVVDDDN---- 40

PF07_0050_D2 -----N**NPC**AKPSGNYPAL-----------**A**HN**VA**YQMH**E**V**A**KTQ**L**RTRGG--------- 35

PFL1950w_D2 ------**NPC**SGDTSGDSNKQYEAV------**A**NT**VA**QILQ**G**K**A**QKQ**L**HGNG---------- 38

PFF0845c_D2 ------**NPC**AVGKKLTKT------------**V**KQ**IA**RQMH**Q**A**A**KKQ**L**GSSSS--------- 33

******* **.** **:* . . .**

PF11_0521_D2 ------GSRSA**L**KGDASQGQ**Y**DRGGKADDFK-T-KL**C**E**I**N-EK**HS**------------------- 68

var16 -------GLSK**L**RAHAEKGE**Y**AL-RGKSSVLND--V**C**K**I**T-LK**HS**------------------- 69

var13 -------GLAK**L**RADAKQGI**Y**KKNRKPQELSN---I**C**N**I**T-LQ**HS**------------------- 69

Var14 -----AGGRKT**L**RADASQGH**Y**NG-KANESVLKD--V**C**D**I**T-NQ**YS**------------------- 66

Var31 -----KGAHNS**L**VGDISKAY**F**KNGGQGSDLKGD-KI**C**D**I**N-TS**HS**------------------- 77

var27 ------AGRSA**L**RGDISLAQ**F**KNGRNGSTLKG--QI**C**K**I**N-EN**YS**------------------- 70

Var1 -------GLSK**L**RAHAHLGT**Y**KR-GGTGNNFKK--L**C**S**I**T-ER**GS**------------------- 69

var12 ------GVSSK**L**KGDAAKGE**Y**RKSGTTIKLKD---I**C**S**I**T-ND**HS**------------------- 68

var17 ----------A**L**KADASKGE**Y**RKRGKADELNN---I**C**K**I**N-PQ**HS**------------------- 67

var44 ----------V**L**KGDISKAT**F**KSGANPSELKN---V**C**Q**I**T-EK**HS**------------------- 73

Var2 ----GKSGNSC**L**VGDITLAK**F**GKAAKSSGLNN-GKF**C**Q**L**DKNK**HS**NAERK-------------- 87

Var7 -------GLYK**L**KGKAHEGI**Y**KRGGRAKDFKYR--L**C**K**I**G-KN**HS**------------------- 75

var8 ---------SK**L**KADAKLGQ**Y**SKKDVERTLN---TE**C**D**I**S-LQ**HS**NR----------------- 73

var11 GKGGGKNVKSS**L**IGDISKAQ**F**KDGTKANGLTE--EV**C**D**I**T-DR**HT**------------------- 86

Var19 ------GGESN**L**KGDASKGK**Y**TKKNGKAVALND--I**C**S**I**D-VQ**HS**------------------- 79

var15 -------SRSA**L**KANASQGK**Y**SNQGDPDDFKKN--L**C**G**I**T-QK**HS**------------------- 67

Var20 ------GGEIN**L**KGDATKGT**Y**RQ-GGPADGFKN-V**C**S**I**N-QN**HT**NVQ----------------- 72

var6 ------GGESN**L**KGDASKGT**Y**GQ-GGHGDVFK-DV**C**N**I**T-AS**HS**-------------------- 69

var10 --------KSS**L**EGDIKKAQ**F**KNGRSGSELNG-DI**C**K**I**D-NK**YS**-------------------- 74

PF08_0141_D2 ---GTENGESV**L**KGDIKKAK**F**KNGASPSSLE--DV**C**G**I**T-DQ**HT**KDSRRRRRLRRLRLLVLRFR 104

PF11_0008_D4 -KVKDSTVESV**L**RADASKGE**Y**KHEGNPDDLKHN-M**R**N**I**T-KE**HT**-------------------- 79

PF11_0521_D3 ---------PG**L**TADAKLGQ**Y**YQNGKVNTLN--NE**C**D**I**S-LE**HS**NR------------------ 60

PF13_0003_D2 ----------K**L**EGDIFKVT**F**RNGGVGKNLNG-DI**C**K**I**D-KT**YS**-------------------- 76

PF13_0003_D5 DKGESSSGKSS**L**EGDISLAE**F**KNGFNPSGLKN--V**C**Q**I**T-EK**HS**YA------------------ 85

PFD0020c_D2 ------*GGEIN****L****KAD****A****SQGK****Y****IRGGKEKKLNGQ-****IC****N****I****D-TS****YS***-------------------- 69

PFD1235w_D3 *SSNGNDKDESK****L****KGK****A****EEGD****Y****SRGGTPSDFNNN-****LC****G****I****T-QK****HS***-------------------- 75

PFD1235w_D2 ------*GGESK****L****KGD****A****TRGT****Y****NLGGQGNTLNGD-****IC****K****I****T-KN****HT***-------------------- 69

PF08_0140_D2 ------GGESN**L**KGDATRGT**Y**NLGCQGDQLDAT-F**C**N**I**D-EK**YS**-------------------- 69

PFF0010w_D2 ------AGEIN**L**KGDATKGK**Y**TKKNGKAVALND-I**C**S**I**D-VQ**HS**-------------------- 86

PFF1580c_D2 ------AGRSL**L**RANASKGE**Y**KHEGNPDDFKKEKL**C**E**I**T-AK**HS**-------------------- 70

PFL0020w_D2 ----------K**L**EGDIFKVT**F**RNGGKGSELQGENI**C**N**I**N-TT**HS**G------------------- 74

PF07_0050_D2 --------RNA**L**RANAAEGK**Y**KNNNKEFTFNG-NI**C**S**I**N-EN**HS**NC------------------ 71

PFL1950w_D2 -------SRNA**L**KGNIQNAK**I**NNGRKPNPLTD--A**C**Q**I**T-KN**HS**-------------------- 72

PFF0845c_D2 ----------A**L**KAHAHLGT**Y**KHTNKRDDFKT--I**C**K**I**T-KD**HS**-------------------- 64

***** **:**   **:*** *** ::**

PF11_0521_D2 **N-**--AR-SNSLN**PC**N**GK**D--NNKVRFN**VGTPW**Q--SGEKIA**T**A-T**DV**YL**P** 109

var16 **N-**--RNPAQSTG**PC**N**GK**D--GDNKRFK**IGTPW**K--GGEQVS**T**SYS**DV**FL**P** 112

var13 **N-**-DSRNGNNGGA**C**T**GK**DGNNE--RFK**IGTEW**K--IGEKVE**T**TDT**DA**YI**P** 113

Var14 **N-**---AIGDSKD**PC**N**GK**---GDG--FK**IGTPW**TN-IVKKKT**T**SYK**DV**FL**P** 106

Var31 **N-**-DSR-GNGGG**PC**I**GK**DGNQGGDRMK**IGTPW**S--KVGEDK**T**TYS**DV**YL**P** 122

var27 **N-**-DSR-GNSGG**PC**T**GK**DGDHGGVRMR**IGTEW**SN-IEGKKQ**T**SYKN**V**FL**P** 116

Var1 **N-**-DSR--TDGE**PC**K**GK**D--GSGVRMK**IGTPW**S--KVGQNK**T**SYS**DV**FL**P** 111

var12 N---AKRGHTDQ**PC**KR**K**D--SKSEMFR**T**E**DGW**K--PAGFIS**K**TYK**D**IYM**P** 111

var17 N--DSRNGNNGE**PC**EG**K**DGSNK--RFE**IGTKW**ET-GGTVQMTE-TEAYM**P** 111

var44 N----ATGESNN**PC**NG**K**GDG-----LQ**IGETW**ED-E-NSKS**N**TL-GMHI**R** 111

Var2 N----RAYTYQG**PC**TG**K**----NQERFK**IGTDW**KD--GDFVSTTHKE**V**FM**P** 127

Var7 N---RDPKRSDG**PC**YG**K**N--EH--RFE**IGKVW**S--HVNEKKTTYT**DV**YL**P** 116

var8 N-----TSRSSG**PC**TG**K**----NEHRFE**IGTEW**KT-GKDVKMTE-NEAYM**P** 112

var11 N--ESRSAPNGG**PC**KN**K**GKG-----LD**IGTKW**N—-DRTSQSST-PN**V**YV**R** 126

Var19 N----STYRSVK**PC**AG**K**----NTGRFD**IGTPW**SYGEKKKEMTH-P**KV**YM**P** 120

var15 N----AIGDSKN**PC**NN**K**GK----ERFN**VGEKW**KN-GGEVKMSH-T**D**LYL**P** 107

Var20 N--NNRAYTYQG**PC**TG**K**DGSNGGVRMK**IGTPW**KP-GRQIQMSA-E**D**IYM**P** 118

var6 N---ATTTFSKG**PC**YG**K**DKVINGVRMK**IGNRW**TH-LQNAK-TTYS**DV**FL**P** 114

var10 N--DIRGSTAGG**PC**TG**K**DGGNE--RFNA**GTKW**EG-DNFVSATH-KNLYI**P** 118

PF08_0141_D2 RRLRRGHRDYKG**PC**TG**K**--DGHKKMFQ**V**E**KGW**EN--GSKIGTE-N**DV**FL**P** 149

PF11_0008_D4 NYQKRGGYNYRG**PC**TG**N**G-NGKDTRFV**IGTIW**KD-EDEKDET--I**KV**LL**P** 125

PF11_0521_D3 N-----TSRSQR**PC**YG**K**--DGYDKMFK**IENGW**KS-GTDINK**K**HPH**DV**FL**P** 102

PF13_0003_D2 N--DSRGTPTDG**PC**EG**K**GD-----RFK**IGTDW**QG-DSFVNP**Q**YR-GIYM**P** 117

PF13_0003_D5 N------GASKD**PC**NG**K**G-NGKDQRFK**I**E**TQW**KD--TGKSG**K**H-V**DV**YL**P** 125

PFD0020c_D2 *N--DSR-N*NNGG**PC**TG**K**NDK----RFK**IGTEW**SYGEHEKKRTH-PE**V**YM**P** 111

PFD1235w_D3 N---AHNDSQQ-**PC**YG**K**DQ----KRFN**VGTEW**SFKDNHRKRTH-PEAYM**P** 116

PFD1235w_D2 *N*--*DSR--P*NGE**PC**TG**K**DKVKNGFRLK**IGTPW**TNIVQKKKK**K**SYK**D**FYL**P** 114

PF08_0140_D2 N---RIPRKSEG**PC**YG**K**----NPQRFY**TGKDW**TH-VVQEK-TSYK**DV**FL**P** 110

PFF0010w_D2 N----STYRSEK**PC**AG**K**----NTGRFD**IGTPW**KT-GTNVKMTE-DQAYM**P** 126

PFF1580c_D2 N--DSRRD--GE**PC**KG**K**DGNNE--RFK**IGTEW**K--IGEKVETSYK**DV**FL**P** 112

PFL0020w_D2 N--DSRGSK-GE**PC**KG**K**DGSGE--RMK**IGTEW**SY-IKEKEIS-YK**D**FYL**P** 117

PF07_0050_D2 N-----HNYSSG**PC**GG**K**--DGRNEMFE**VKDGW**KS-GADVSK**E**HAE**DV**FL**P** 113

PFL1950w_D2 N----GKGDSNN**PC**NN**K**G-----NRLK**IGQVW**S----IKNDTSYT**DV**YM**P** 109

PFF0845c_D2 N---ATHNYPQG**PC**HG**K**N--NDKSMFK**TEEGW**K--PGNQIN**M**NDE**Y**AFM**P** 107

**Loop 1**

**.*** ***** :  ***** :: *****

PF11_0521_D2 **PRR**Q**H**F**CTSNLE**Y**L**INGGHQAILNVKNGKIN------HSF**L**GD**V**LLA**A**KYQAQHTMKD**Y**K 163

var16 **PRR**Q**HMCTSNLE**H**L**NTKSTGLSESK---------LASNSL**L**GD**V**LLA**A**KYEAEDIKKN**Y**K 163

var13 **PRR**Q**HMCTSNLE**N**L**NVS----WVTEDG-------KAIHSL**L**GD**V**QLA**A**KMDADEIIKR**Y**K 162

Var14 **PRR**E**HMCTSNLE**N**L**DVG--DVTNNV---------NVNNKF**L**VQ**V**LLS**A**NKQAEWIKQK**Y**N 155

Var31 **PRR**Q**HMCTSNLE**F**L**ETKDTPLDGKFGVDKIN------HSF**L**GD**V**LLV**A**NFEAKNIKEL**Y**K 176

var27 **PRR**E**HMCTSNLE**N**L**DVG----SVTKND-------KASHSL**L**GD**V**QLA**A**KTDAAEIIKR**Y**K 165

Var1 **PRR**E**HMCTSNLE**K**L**EVD--WVTNKG---------KASHSL**L**GD**V**LLS**A**KMDAEKIIQL**Y**K 160

var12 **PRR**Q**H**F**CTSNLE**Y**L**QTTNKLLNGNDINGNPN---IINDSF**L**GD**V**LFA**A**NYEADFIKKMYK 168

var17 **PRR**Q**HMCTSNLE**N**L**DVD----SVIKND-------KASHSL**L**GD**V**LLS**A**NHEAKKIKELYE 160

var44 **PRR**K**HMCTSNLE**K**I**NVG----NVTQNG-------NINPSF**L**VD**V**LLA**A**KEEAEDIKKKYK 160

Var2 **PRR**E**HICTSNLE**N**L**DIKSE----GLSNG-----SFSSHSL**L**GD**V**FLS**A**KYEAENIKKLYQ 178

Var7 **QRR**E**HMCTSYLE**Y**L**QTNISPLNGMEIVKNGKNGKLVNDSF**L**GD**V**LLS**A**KFEGDYILKNFN 176

var8 **PRR**Q**HMCTSNLE**Y**L**INGNHKEILKIENG-----KIN-HSF**L**GD**V**LLA**A**KKEAEFIKSKVT 166

var11 **PRR**E**HMCTSNLE**H**L**ETDQGPLNKSDGK-------VVNDSF**L**GD**V**LLS**A**KYEAEKIKELYE 179

Var19 **PRR**E**HMCTSNLE**Y**L**ETGEGPLKGKDNN-----GKLVNNSF**L**GD**V**LLS**A**KKEG----DFIV 171

var15 **PRR**Q**HFCTSNLE**H**L**NTKS----TGLT------SDKAIHSL**L**GD**V**LLA**A**KKEGEDIKTKLT 157

Var20 **PRR**Q**HMCTSNLE**Y**L**QTKDGPLKQGDG-------KLVNNSF**L**GD**V**LLS**A**KMDAGKIIELYK 171

var6 **PRR**Q**HMCTSNLE**Y**L**QTKDGPLNGMEIVKNGKNGKLVNNSF**L**GD**V**LLS**A**KMDAGKIIELYK 174

var10 **PRR**Q**HMCTSNLE**K**L**DF----LSVTSKS-------NVNDSF**L**GD**V**LLA**A**NNEAQRTKDHFA 167

PF08_0141_D2 **PRR**E**H**F**CTSN**V**E**H**L**YRSAS----GLQG------TTASHSL**L**GD**V**LLA**A**NKEAGFIKERYK 199

PF11_0008_D4 **PRR**R**HMCTSNLE**Y**L**LHVNKGPLLKVEPD-----KIN-HSF**L**GD**V**LLA**A**KYEAEFIKTNYT 180

PF11_0521_D3 **PRR**E**H**F**CTSNLE**H**L**NTNVS----GLTG------PNAIHSL**L**GD**V**LLA**A**KKEAGFIEEKYK 152

PF13_0003_D2 **PRR**Q**H**F**CTSNLE**K**L**DVS----RVIRNG-------NASNSL**L**GD**V**LLA**A**KYEAERTKNHYV 166

PF13_0003_D5 **PRR**E**H**I**CTSNLE**Y**L**LKGNSDQIMKVGNN-----KIN-HSF**L**GE**V**LLA**A**KYEAEFIKTNYT 179

PFD0020c_D2 **PRR**E**HMCISNLE**K**L**DVVS-VIKNGN----------ASHSL**L**GD**V**LLA**A**KYEAKNIKELYQ 160

PFD1235w_D3 **PRR**E**H**I**CTSNLE**Y**L**IHKRKKPIIEGD------PNKIIHSL**L**GD**V**LLA**A**KYEAENIKKLYE 170

PFD1235w_D2 **PRR**Q**HMCTSNLE**N**L**STSSK----GLSNGSFAS-----HSL**L**GD**V**LLA**A**KFEAQKIILVYK 165

PF08_0140_D2 **PRR**E**HMCTSNLE**N**L**NLNSKGLSNSS---------IASNSL**L**GD**V**LLA**A**KYEAD---DIVK 158

PFF0010w_D2 **PRR**E**HMCTSNLE**Y**L**ETDQGPLKNSD-------GKFVNHSF**L**GD**V**LLA**A**NHEAKKIKELYT 179

PFF1580c_D2 **PRR**Q**HMCTSNLE**N**L**DVD----SVTEND-------KASHSL**L**GD**V**QLA**A**KTDAAEIIKRYK 161

PFL0020w_D2 **PRR**Q**HMCTSNLE**N**L**DVE----SVTKED-------KASHSL**L**GD**V**QLA**A**KYEAENIKKLYV 166

PF07_0050_D2 **PRR**Q**H**F**CTSNLE**N**L**NTNSK----GLSDG-----TLASHSL**L**GD**V**LLS**A**NKEAGFIKDKYK 164

PFL1950w_D2 **PRR**Q**HMCTSNLE**K**L**NYAS---VIG--------SNNVNDKF**L**VE**V**LHA**A**KSEAEFIKKKYN 158

PFF0845c_D2 **PRR**Q**H**F**CTSNLE**Y**L**ETADRTLNG--IGDDPN---VLNHSF**L**GD**V**LLA**A**KFEADFIKEKYN 162

******.***:* * :*** **:** .:***** :*** ***: :.

PF11_0521_D2 SKN------------DKEGI**CRA**IR**YSFAD**I**GDII**K**G**T**DLWD**KDG**G**EIKTQNH**L**VT**IF**D 210

var16 ERNG---Q---ID---NKGK**CRA**IR**YSFADLGDII**R**G**R**DLWD**LDE**G**SKKMEGH**L**KK**IF**K 213

var13 KHN---TLTDPIQQKDQESI**CRA**VR**YSFADLGDII**R**G**R**DLW**EHG-DQTKLQGH**L**QI**IF**G 217

Var14 EPNG---Q------NNHKGK**CRA**LK**SSFADLGDII**K**G**T**DLWD**KDS**G**EQKTQRN**L**VT**IF**G 205

Var31 NNND---RKDLNDANDKETV**CRA**MK**YSFAD**I**GDII**R**G**T**DMWD**KDE**G**SKKMDVI**L**KK**IF**G 232

var27 DQNNI-QLTDPIQQKDQEAM**CRA**VR**YSFADLGDII**R**G**R**DMWD**EDKSSTDMETR**L**ITV**F**- 222

Var1 EHKK---KDELTDPNYQETV**CRA**IR**YSFADLGDII**R**G**R**DMWD**LND**G**SQKIEKN**L**KD**IF**D 216

var12 KQND---------YKDNATI**CRA**MK**YSFADLGDII**K**G**T**DMWD**NDS**G**ESKTRDK**L**RE**IF**D 218

var17 KNKDQSGQNDKNGLTDDKTV**CRA**MK**NSFAD**I**GDII**R**G**R**DLWD**NK-DQVTLQDH**L**KT**IF**G 218

var44 EIKDKNGLKD-----DQVTT**CRA**IK**SSFAD**I**GDII**R**G**R**DLW**ENG-EAKSLQGN**L**VT**IF**G 213

Var2 QNNSK---IKLTEEKDKESI**CRA**LR**YSFADLGDII**R**G**K**DLWD**-HKDFKKLEKH**L**QK**IF**G 233

Var7 KKN-----------KAVPGI**CRA**MK**YSFAD**I**GDII**R**G**R**DMWD**LDD**G**SKKMEDIFKK**IF**G 224

var8 ------------NNDNGSAI**CRA**MK**YSFAD**I**GDII**R**G**K**DLWD**-HRDFKNLERD**L**VT**IF**G 212

var11 KNKDQS---------GHEVI**CRA**IR**YSFAD**I**GDII**R**G**R**DLWG**RDS**G**STDMETR**L**KN**IF**K 229

Var19 K------KLGS----DKSAI**C**N**A**MK**YSFADLGDII**R**G**R**DMWD**KDS**G**SKKMDVI**L**KNV**F**G 220

var15 ENDN------------RSSI**CR**TMK**YSFAD**I**GDII**R**G**T**DLWD**INGDATGVQNN**L**KD**IF**S 204

Var20 KQNN---KSNLTDPEDNESA**CRA**LR**YSFADLGDII**R**G**R**DLWD**KNSDAKRLQTN**L**KE**IF**T 227

var6 KQNN---KSNLTDPEDNESA**CRA**LR**YSFAD**IA**DII**R**G**R**DMWD**KDD**G**AQKIDVI**L**KNV**F**G 230

var10 HKKDD-----------HGIA**CRS**VR**YSFADL**A**DII**R**G**R**DMWD**KDD**G**AQKMEDIFKK**IF**G 215

PF08_0141_D2 ---TQ---KTSEGFKDEATV**CRA**IK**YSFAD**IA**DII**K**G**T**DLW**KANS**G**KKNTQDK**L**VK**IF**Q 252

PF11_0008_D4 ---------RLNGQNDNGAK**CRA**MK**YSFAD**I**GDII**R**G**K**DLW**G-IQDFKDLQTK**L**VT**IF**G 229

PF11_0521_D3 ---KP---ITPEGFKENVTM**CRA**IK**YSFADLGDII**K**G**T**DLWD**GNG**G**EKNTQSN**L**ET**IF**G 205

PF13_0003_D2 SKKEE-----------HSEA**CRA**VR**YSFADLGDII**R**G**K**DMWD**KNH**G**EKKTQEN**L**ER**IF**A 214

PF13_0003_D5 ---------RLNGQNDNGAK**CRA**MK**YSFAD**I**GDIV**R**G**R**DLW**E-HNDFKKLERD**L**VK**IF**G 228

**Loop 2**

PFD0020c_D2 QNNS---KNGVIDQNDKETI**CRA**MK**YSFAD**I**GDII**R**G**K**DMWV**QNTDATKLQAY**L**AK**IF**D 216

**Loop 2**

PFD1235w_D3 ENNN---------RKDQEGI**CRA**MK**YSFAD**I**GDII**R**G**K**DMWI**ENNDAKRLQTN**L**KE**IF**T 220

PFD1235w_D2 NKNNINIRKRITDPNDQATV**CRA**IR**YSFADLGDII**R**G**K**DMWN**INSDAKDLQDR**L**EK**IF**K 224

PF08_0140_D2 K-------NDS----DKSSI**C**N**A**MK**YSFAD**I**GDII**R**G**K**DMWD**LDS**G**SKDMEKH**L**IS**IF**E 206

PFF0010w_D2 K------DNGLNDLKDKETV**CRA**MK**YSFADLGDII**R**G**R**DMWD**NET**G**MKHAKKH**L**KDV**F**D 232

PFF1580c_D2 DQNNI-QLTDPIQQKDQEAM**CRA**IR**YSFADLGDII**R**G**R**DMWN**KDS**G**STEMEKH**L**IS**IF**- 218

PFL0020w_D2 ENN---------DRKDQEAI**CRA**VR**YSFAD**I**GDII**R**G**R**DMW**EHK-DQTTLQNH**L**KSV**F**- 214

PF07_0050_D2 ---NQ---PTLGGFKDEATI**CRA**MK**YSFAD**I**GDII**K**G**T**DLWD**GNKEETDTQRN**L**VT**IF**G 217

PFL1950w_D2 EKQND---GKNGLRKDQATT**CRA**IR**YSFAD**I**GDII**R**G**K**DLWD**DNNDAKSLQTN**L**KA**IF**K 214

PFF0845c_D2 EQSN---------YKDFSTI**CRA**MK**YSFADLGDII**K**G**T**DLWD**KNG**G**EQKTQGK**L**EK**IF**C 212

*****.::: ******:.**:**:***** ***:***  **:** **:***

PF11_0521_D2 ----K**IK**AQL**P**K**DIK**--G**KY**TG-------T**K**HLE**LR**K**DWWEANR**DQ**VW**K**AM**Q**C**GND------- 253

var16 ----Q**IK**EKH**P**-**GVQ**--E**KY**NSDNDY---N**K**YIN**LR**S**DWWEANR**HK**VW**K**AM**K**C**EISELKDMSG 266

var13 KIKEE**IK**KKH**P**-**GIN**GND**KY**KGDEKNNPPY**K--**Q**LR**E**DWWEANR**HQ**VW**R**AM**Q**C**ELKN------ 271

Var14 ----K**IK**VQR-K**GID**-TS**KY**TNTDG-----**K**HNQ**LR**E**DWWEANR**RQ**VW**K**AM**K**C**ALK------- 250

Var31 ----K**IK**QEL**P**K**EIQ**--K**KY**KNPDG-----**K**HTQ**LR**K**DWWEANR**HQ**VW**R**AM**K**C**AIQ------- 277

var27 ---KN**IK**EKH-D**GIK**DNP**KY**TGDESKKPAY**K--**K**LR**A**DWWEANR**HQ**VW**R**AM**K**C**ATKG------ 273

Var1 ----K**IK**DNL**P**D**GIK**DNRQ**Y**NGDPN---HI**K---LR**E**DWWEANR**KQ**IW**H**AM**Q**C**ALKS------ 263

var12 ----T**IK**KKHP-GIK--E**IY**KEDTP---YT**K---LR**E**DWWEANR**KK**IW**E**AM**Q**C**PTP------- 261

var17 ----K**IK**G----ELKGED**KY**NRDDKKSPPY**K**--Q**LR**A**DWWEANR**DQ**IW**E**AM**I**C**ETKS------ 265

var44 ----H**IH**SS-P--**-**NGKG**KY**ASDEKNNPPY**K**--Q**LR**E**DWWALNR**ET**VW**D**AM**K**C**KTN------- 259

Var2 ----K**IK**EELK**S**KIN--D**KY**EDNSEG----**K**HTK**FR**E**DWWEANR**AK**VW**E**AM**Q**C**PKKIP----- 281

Var7 ----TLHKSL-DGIK--D**KY**KEGEP---YT**K**---**LR**E**DWWEANR**HQ**VW**R**AM**K**C**AIKGLNVKSP 274

var8 ----K**IK**EGITDETIK-E**KY**DSYKDN----**K**HIQ**LR**S**DWWEANR**DQ**IW**K**AM**Q**C**PPKK------ 260

var11 ----K**IK**EQIP-EIH--D**KY**KDDENKTPPY**K**--Q**LR**E**DWWEANR**RQ**VW**N**AM**T**C**PTKN------ 277

Var19 ----TL**H**KSL-EGIRNHP**KY**AYDKNKTSPY**K**--Q**LR**E**DWWEANR**HQ**VW**R**AM**K**C**EIKK------ 270

var15 ----K**I**TEELKK--QHPD**K**FNDNDKYTNDS**K**HTK**LR**S**DWWEANR**DQ**VW**K**AM**T**C**PTKNG----- 256

Var20 ----K**IK**EELPEDIK--K**KY**DKDG---TDH**K**--L**LR**E**DWWEANR**HQ**VW**R**AM**K**C**AIEN------ 273

var6 ----TL**H**KSL-EGIRNHP**KY**AYDKNKTSPY**K**--Q**LR**E**DWWEANR**HQ**VW**R**AM**K**C**ATKA------ 280

var10 ----NLYESLP-GIK--G**KY**DGDDQRTPQY**K**--Q**LR**E**DWWEANR**DQ**VW**K**AM**V**C**E-KD------ 262

PF08_0141_D2 ----K**IK**DNLPVNIK--G**KY**NE------DE**K**HLE**LR**K**DWWFANR**DK**VW**E**AM**K**C**EQ-------- 295

PF11_0008_D4 ----K**IK**EEIP-DIK--K**KY**SSENPP--YT**T**---**LR**E**HWWEANR**AK**VW**E**AM**Q**C**P-TIP----- 274

PF11_0521_D3 ----K**IK**DNL--G-KSVE**NY**KDD--SYPYI**K---LR**S**DWWSANR**DK**VW**K**AM**T**C**PQ-------- 248

PF13_0003_D2 ----K**IK**EQLLNS**SI**K-D**KY**KDDDKATPKY**K**--Q**LR**E**DWWEANR**SQ**VW**E**AM**Q**C**PPKN------ 264

PF13_0003_D5 ----K**IK**EGI-TD**E**TTKK**QY**EK---DDTDN**K**--Q**LR**C**DWWEANR**DQ**VW**E**AM**Q**C**KTTIP----- 276

PFD0020c_D2 ----K**IK**DNH-KDIKGKL**QY**N----GDTDH**K**--L**LR**E**DWWEANR**HQ**VW**R**AM**K**C**AIEN------ 262

PFD1235w_D3 ----K**IK**EKT-----GGT**TY**NE--DNDPYL**K---LR**A**DWWEANR**AK**VW**K**AM**K**C**KT-NGV---- 264

PFD1235w_D2 ----T**IN**EKLPNEIQ--K**RY**TNREN-----**K**HLD**LR**S**DWWEANR**HQ**VW**R**AM**K**C**ATK------- 269

PF08_0140_D2 ----K**IK**ENL-DVIKYNS**KY**KDTQ------**K**FLD**LR**S**DWWEANR**HQ**VW**R**AM**K**C**AIEN------ 252

PFF0010w_D2 ----N**I**RKSL-KN**K**GNQ-**KY**NYDDKKLPPY**K**--E**LR**E**DWWEANR**HQ**VW**R**AM**K**C**AIKE------ 281

PFF1580c_D2 ---EK**I**NEKLPEK**E**QK--**KY**SNDG------**K**YLD**LR**K**DWWEANR**YK**VW**K**AM**K**C**ATKN------ 264

PFL0020w_D2 ---KN**IK**EKLP-GIQG--**KY**ADDERNIPAY**K**--L**LR**E**DWWEANR**RQ**VW**K**AM**T**C**ENNG------ 263

PF07_0050_D2 ----K**IK**DKI-RD**E**ATKK**KY**SD------AQ**K**HLQ**LR**K**DWWEANR**DQ**VW**K**AM**Q**C**G--------- 260

PFL1950w_D2 ----K**IK**EKHP-GIEGND**KY**VKDN----EN**K**--Q**LR**S**DWWEANR**RQ**VW**N**AM**T**C**ETPNGD---- 262

PFF0845c_D2 ----K**IK**NKLPEDIQ--E**KY**INDDKNSPQY**K--**K**LR**E**DWWEANR**KE**VW**R**AM**T**C**ATT------- 260

**:** ***** ******.*********** ****** ****** *****

**Loop 3**

PF11_0521_D2 -----NP**C**-SGESDHT**P**LH**DYIPQRLRWMTEWAEWYCK**E**Q**SRL**YD**K**L**K 295

var16 HHASSSH**C--**GYSHGM**P**VD**DYIPQRLRWMTEWAEWYCK**A**Q**SQE**YD**K**L**M 312

var13 LKKSNGD**C**-HYNSRGT**P**LD**DYIPQRLRWM**V**EWAEWFCK**M**Q**SQE**YD**K**L**M 318

Var14 --GEKIN**C**-----GAT**P**YD**DYIPQRLRWMTEWAEWFCK**E**Q**SRL**YD**E**L**M 291

Var31 -DGSIEK**C**-----NGI**P**LD**DYIPQRLRWMTEWAEWFCK**M**Q**KEA**Y**NE**L**K 319

var27 -----II**C-----**PGM**P**VD**DYIPQRLRWMTEWAEWYCK**A**Q**SQE**YD**K**L**K 311

Var1 --GNEIQ**C**----NNHT**P**IE**DYIPQRLRWM**N**EWAEWYCK**E**Q**SML**Y**NK**L**V 305

var12 --NGSFP**C**---KSYHT**P**LD**DYIPQRLRWMTEWAEWFCK**E**Q**KKQ**Y**GE**L**V 304

var17 --HPTIK**C----**DKTT**P**YD**DYIPQRLRWMTEWAEWYCK**A**Q**TEA**Y**GE**L**L 307

var44 --GVDIT**C----**DSDV**P**FD**DYIPQGLRWMTEWAEWYCK**A**Q**AEA**Y**GE**L**L 301

Var2 PPGVDIK**C---**DQTGV**P**LD**DYIPQRLRWMIEWAEWYCK**Y**Q**SQE**Y**EK**L**K 326

Var7 DGKLSDH**C**--GYSDHT**P**LD**DYIPQRLRWMTEWAEWYCK**E**Q**KKQ**Y**HD**L**V 320

var8 ---AIFP**C**-RDNKDTV**P**LD**DYIPQRLRWMTEWAEWFCK**M**Q**KKA**Y**EQ**L**E 304

var11 ----GIT**C-----**DGS**P**YE**DYIPQRLRWMTEWAEWYCK**M**Q**SQE**Y**EN**L**V 316

Var19 --DKNMK**C**-----NGI**P**IE**DYIPQRLRWMTEWAEWYCK**A**Q**NKY**Y**GE**L**E 311

var15 ----NIQ**C-----**GAT**P**HD**DYIPQRLRWMVEWAEWFCK**E**Q**SRL**Y**EE**L**L 295

Var20 --DKDMK**C**-----NGI**P**IE**DYIPQRLRWMTEWAEWFCK**E**Q**SRL**Y**NK**L**V 314

var6 --IPDMK**C**-----NGI**P**IE**DYIPQRLRWMTEWAEWFCK**M**Q**SQE**Y**EK**L**E 321

var10 ----GIK**C---**DEDPT**P**VD**DYIPQRLRWMTEWAEWYCK**V**Q**SQE**Y**DE**L**L 303

PF08_0141_D2 ---NGIT**C**----SGPT**P**LD**DYIPQRLRWMTEWAEWYCK**A**Q**KEA**Y**DK**L**K 336

PF11_0008_D4 --PVTTS**C**--DTTTVT**P**LV**DYIPQRLRWMTEWAEWFCK**M**Q**SQE**Y**EV**L**V 318

PF11_0521_D3 ---NGIK**C**----DKDP**P**LD**DYIPQRLRWMTEWAEWYCK**Y**Q**AEA**Y**KT**L**Q 289

PF13_0003_D2 ---GTFP**C---**KSDHT**P**LH**DYIPQRLRWMTEWAEWYCK**E**Q**SRL**Y**GE**L**V 306

PF13_0003_D5 --PVTTS**C**--DTTTVT**P**LV**DYIPQRLRWMMEWAEWYCK**Y**Q**SKA**Y**SE**L**R 320

PFD0020c_D2 --DKDMK**C**-----NGI**P**IE**DYIPQRLRWMTEWAEWYCK**E**Q**SRL**Y**GE**L**L 303

PFD1235w_D3 ----DIT**C**---DSDHT**P**LD**DYIPQRLRWMTEWAEWYCK**A**Q**SQE**Y**KK**L**E 305

PFD1235w_D2 -GISNNN**C**-----NGI**P**IE**DYIPQRLRWMTEWAEWYCK**K**Q**SQE**Y**EK**L**E 311

PF08_0140_D2 --DKDMK**C**-----NGI**P**IE**DYIPQRLRWMTEWAEWYCK**A**Q**KKE**Y**DE**L**V 293

PFF0010w_D2 --ATIDN**C**-----NGI**P**IE**DYIPQRLRWMTEWAEWYCK**M**Q**SQE**Y**KK**L**Q 322

PFF1580c_D2 ---SKIP**C-----**SGI**P**IE**DYIPQRLRWMTEWAEWFCK**E**Q**SQA**Y**ET**L**Q 304

PFL0020w_D2 -----IK**C**DAHNAKHP**P**PD**DYIPQRLRWMTEWSEWYCK**M**Q**SQE**Y**EK**L**K 306

PF07_0050_D2 ---NDNP**C**--SGVSGV**P**LD**DYIPQRLRWMTEWAEWFCK**M**Q**SQE**Y**NK**L**M 303

PFL1950w_D2 ----NIK**C**---DVHDV**P**VD**DYIPQRLRWMTEWAEWFCK**A**Q**SQE**Y**DK**L**F 303

PFF0845c_D2 --SGKIP**C**----SIVT**P**LD**DYIPQRLRWMTEWSEWFCK**E**Q**SKL**Y**GE**L**V 302

***** ***** ******* **** **:**:** *** ***** *****

PF11_0521_D2 V-**C**EE**C**MRKGE----S**C**TKGSGE----**C**AT**C**KE**AC**EE**Y**NKEIKK**W**EQ**QW**DAISY 340

var16 GA**C**GS**C**MGKGK--VQG**C**TSGDVDSVKK**C**EK**C**KT**AC**DE**Y**WNKIKP**W**KG**QW**NTMEI 364

var13 KQ**C**SQ**C**MSKG----GD**C**RKGDVN----**C**TS**C**EQ**AC**EE**Y**KKKIKK**W**EK**QW**NKIKD 364

Var14 GK**C**GI**C**MN-GI-----**C**NKVKDD----**C**AK**C**TE**AC**KE**Y**KTKIQP**W**KD**QW**EKLEL 335

Var31 GK**C**SQ**C**KTKDK----K**C**TNKSDD----**C**NT**C**TE**AC**TA**Y**NRKINT**W**KQ**QW**DAISD 365

var27 KISAD**C**MSKGD---GK**C**TQGDVD----**C**GK**C**KA**AC**DK**Y**KEEIEK**W**NE**QW**RKISD 358

Var1 AD**C**GD**C**MKKGE-GGKE**C**MNGSGE----**C**RK**C**KQ**AC**EE**Y**KAKIKK**W**QE**QW**DKMQL 354

var12 SA**C**NG**C**KDEGK----V**C**TNKSSQ----**C**TS**C**MQ**AC**EN**Y**KNFINT**W**KE**QW**DKMEI 350

var17 RD**C**GS**C**TGKVQ-----**C**KEGTKE----**C**KD**C**KSS**C**EK**Y**TEFVNK**W**KP**QW**NTMEI 352

var44 QK**C**GN**C**KDKIKG-HGQ**C**KEGNKE----**C**TV**C**KSS**C**KK**Y**TEFVNK**W**KP**QW**NKMDM 350

Var2 RG**C**EG**C**RSKGGQ----**C**KNGESM----**C**NS**C**TK**AC**NT**Y**KENINK**W**QK**QW**KQIKQ 372

Var7 NK**C**KE**C**KKKDNGG--H**C**WKDSAE----**C**TE**C**DE**QC**KK**Y**KKFIDT**W**QP**QW**DKIRA 368

var8 RK**C**GE**C**RS--GK----**C**ET-EKN----**C**KE**C**KA**KC**KE**Y**KEKITP**W**KQ**QW**EKMSK 347

var11 NV**C**NG**C**KDKG----DD**C**RNNSAE----**C**SP**C**KE**AC**EA**Y**RDKIKK**W**EE**QW**QQMQL 362

Var19 KE**C**GG**C**MG----NGQG**C**TSSDPK----**C**KQ**C**KN**AC**DA**Y**KKKIET**W**EK**QW**EKIKG 357

var15 RD**C**GS**C**TTG------K**C**NNDK------**C**AK**C**DK**QC**QE**Y**KTKIQP**W**AD**QW**NEISN 337

Var20 AD**C**KS**C**KG----KAKS**C**TQKDGD----**C**TK**C**KA**AC**DN**Y**NKKIKP**W**EE**QW**EKIKN 360

var6 KE**C**GG**C**KE----MGGQ**C**TNDTED----**C**QR**C**TQ**AC**EE**Y**KKKIEP**W**EK**QW**TKIKD 367

var10 KK**C**GS**C**KIKGK--VQG**C**TSGDSD----**C**TP**C**AE**AC**TT**Y**GQKIKP**W**ED**QW**NNMLL 351

PF08_0141_D2 V-**C**EK**C**MDNGK-----**C**TQGNGE----**C**AK**C**KT**AC**EN**Y**KKFINT**W**QP**QW**KQMEQ 380

PF11_0008_D4 KQ**C**RN**C**RS-GI-----**C**ENGKDD----**C**VK**C**TQ**AC**NT**Y**KQKIKK**W**ED**QW**KEISK 362

PF11_0521_D3 KG**C**DE**C**KNKSKK----**C**EKDKPQ----**C**KN**C**TK**AC**DE**Y**WKKIEK**W**EN**QW**TKIKE 335

PF13_0003_D2 ET**C**GK**C**MHKG-----K**C**KQGNGH----**C**VT**C**KP**AC**EK**Y**KKFINT**W**QP**QW**KQMEQ 351

PF13_0003_D5 KG**C**ED**C**RS--WK----**C**MKGDSK----**C**EN**C**TK**AC**KD**Y**NSKIEP**W**KQ**QW**TKIKE 364

PFD0020c_D2 EK**C**QS**C**KG-----KQK**C**TEGDVD----**C**GK**C**KA**AC**DK**Y**KDEINK**W**RE**QW**TKIKG 348

PFD1235w_D3 EK**C**SQ**C**KSKGK-GGNE**C**YRETKE----**C**ND**C**KQ**AC**EE**Y**KRKIKT**W**AD**QW**KVISN 354

PFD1235w_D2 EK**C**GM**C**TGKGQGDGKD**C**TQKDKE----**C**SP**C**KK**AC**DA**Y**KKEIEK**W**EK**QW**KTVSA 361

PF08_0140_D2 KG**C**KG**C**MG----NGQG**C**TKDSSDG--E**C**KK**C**TE**AC**NT**Y**KQKIKT**W**EN**QW**KKIKG 341

PFF0010w_D2 DA**C**TG**C**KK----KVDS**C**TKGTPD----**C**EQ**C**DK**QC**KQ**Y**TEFITK**W**QP**QW**ETMSY 368

PFF1580c_D2 DQ**C**GK**C**TGPNK---DN**C**TRDNND----**C**NT**C**TK**AC**EE**Y**EQKIKK**W**AD**QW**KVISK 351

PFL0020w_D2 EG**C**KK**C**MENKG---KN**C**IKDTPE----**C**ND**C**KQ**AC**EE**Y**NKKIKE**W**EK**QW**HKIQV 353

PF07_0050_D2 EA**C**TG**C**MKKGKDG-EG**C**TQKTQE----**C**AL**C**KA**AC**DA**Y**KKEIEK**W**QR**QW**NNMQV 352

PFL1950w_D2 MQ**C**AK**C**MGNGQ----G**C**TKDSSD--GE**C**EK**C**KE**AC**TS**Y**ANFINT**W**KP**QW**VPMQI 351

PFF0845c_D2 KD**C**AS**C**KKKGKE---K**C**TQGDND----**C**TP**C**DK**KC**KE**Y**GKKIRT**W**KD**QW**TKMDG 349

**.** ***** ***** *** ***  ***** *****  : *** **** :

PF11_0521_D2 **KY**LM**LYA**K**A**RI-TAI**N**G**G**PGYYNT-EVQEEDKP**VV**D**F**LYN**L**YLQNGGKKGPPPDTHRVKALIARVKR 405

var16 **KY**LT**LYA**Y**A**QM-ASN**N**K**G**DMSIF**G**NAVGPKDKP**VV**Q**I**LQE**L**LP----PKSVKP-------GAPT--- 416

var13 **KY**EE**LYL**Q**A**KI---A**F**A**G**--TSF**G-**GGDRDYQQM**V**H**F**FKE**L**QKV---------------TGDTTLG- 409

Var14 E**Y**ALS**YL**H**A**KN-DSRRMA----F**G-**GTDPDYQQ**VV**H**F**FKE**L**QEAIKSSTSKRPKRST------DAIT 390

Var31 **KY**QF**LYL**Q**A**KT-AAA**N**G**G**PHASS**G-**DVGEKDKP**VV**N**F**LFE**L**YKQNGGKISTPSDTHPG----PRVKR 426

var27 **KY**NL**LYL**Q**A**KT-TST**N**P**G**RT-VL**G-**DDDPDYQQM**V**D**F**LTP**I**HKASIAARVLVKRAAGSPTEIAAAA- 421

Var1 **KY**LYF**YH**E**A**KT--TSRH**G**IDAYS**G-**AVEPKDKP**VV**K**F**LQE**L**LP----PKSVKP----------GAPT 404

var12 **KY**KL**LY**LQ**A**QT-TAA**N**G**G**PDTYSG**-**LVDENEKP**VV**N**F**LFE**L**YKENGGKIGNPRDTPRAKRSKRETAP 415

var17 **KY**VP**LY**LQ**A**KN---A**Y**Y**G**IS--FP---GADYQLM**V**D**F**LSK**L**HTSS------VAAIGKNGET--TNS- 403

var44 **KY**TP**LY**LQ**A**KN---P**Y**P**G**IV--FP---GADYQLM**V**D**F**LSK**L**HTEN------IAASSKSSTTRVTATP 403

Var2 **KY**DD**LYQ**K**A**KQ----**N**GV-------TSSDKDAD**VV**R**F**LKQ**L**HKENGGGKSGATT------------- 415

Var7 **KY**KK**IYE**H**A**RVDIAA**N**G**G**LNT-STAINDNEDKP**V**IE**F**LFE**L**YKANGGKISNPAVARATVNG----IS 430

var8 **KY**KQ**LYE**Q**A**KK--DT**N**CSTL-------TKQDQDA**V**A**F**LKK**L**KNQN-----TANE------------- 387

var11 Q**Y**LI**LYH**L**A**NT--TGPH**G**INSYGG-AVGEKDKP**VV**Q**F**LEE**L**QKANG----------------VAASD 410

Var19 **KY**EE**LYK**K**A**LDSVNG**N**GKGEK-STTSGTKDEKD**VV**D**F**LKQ**L**LAQNSAAARVRVIRAAGSPT--EITA 421

var15 **KY**QI**LYW**Q**A**KI-AAI**N**G**G**TEK-STTTKDDKDKN**V**ID**F**LQK**L**HEANYGTRGPPPEAHPDRRP--RRAA 400

Var20 **KY**AQ**LYK**K**A**LDSVNGKEES--KKKTASDAKDQQ**VV**H**F**LAE**L**IRKSGGGKGGKNVKTT------VSPT 419

var6 **KY**KT**LYE**Q**A**TK-NGETS**G**TP-------NEKDKD**VV**D**F**LKQ**L**LAQNSAAARNRVIRAAGNSATRVIAT 426

var10 Q**Y**TL**LYW**Q**A**ET-TARYG**G**TRAYSG-DVGDKDKP**VV**Q**F**LEE**L**QKQN---------------------- 394

PF08_0141_D2 **KY**ES**LYK**E**A**QE--NG**N**SSH-----KSTTEQDKYM**V**E**F**LSQ**L**QKANNGDKTGDDK------------- 427

PF11_0008_D4 **KY**KT**LYQ**Q**A**KG--SV**N**GATTS---STTDEKDKD**VV**D**F**LKM**L**HQKN-----TDNT------------- 406

PF11_0521_D3 **KY**EQ**LYQ**K**A**EK----**N**D**G**DTS-SGTDDVKEEND**VV**A**F**LSQ**L**YEKNKGSNT----------------- 380

PF13_0003_D2 **KY**SQ**LYE**E**A**KK---Y**N**DSS----RKDTTNKDDY**VL**Q**F**LNK**L**LTQN---------------------- 389

PF13_0003_D5 **KY**EE**LYK**K**A**QNSDTS**N**S**G**TT-Y-----PKDEKD**VV**S**F**LSK**L**HEKN-----KDNK------------- 407

PFD0020c_D2 **KY**KT**LYK**K**A**TK-PGVTTSN-------NPKDEKD**VV**D**F**LKQ**L**LPR-------KSKNTP------GVTA 394

PFD1235w_D3 **KY**ED**LYK**K**A**QN--PT**N**AVL----KDNKDEKDKN**V**ID**F**LTQ**L**QKANN------------------GEK 397

PFD1235w_D2 **IY**QI**LY**AK**A**RI-VAS**N**G**G**PGYY-NTEVQKKDRS**VY**D**F**LYE**L**HLQNGGKKGPPPATHPYKSVNTRDKR 426

PF08_0140_D2 **KY**EK**LYE**Q**A**TK-NGETS**G**TP-------NEKDKD**VV**D**F**LKQ**L**LPRNSAAARNRVIRAAGSSAP-GVTA 399

PFF0010w_D2 **KY**QT**LYE**E**A**ER--DATS**G**SVK-KRTQLSKEDQR**VV**D**F**LKQ**L**LLRNSAAARNRVIRAAGSSATGGTTA 432

PFF1580c_D2 **KY**EE**LY**LQ**A**KT-AFARTA---F--PDDDPDYQQ**VV**E**F**FKE**L**QKE--INRSASQRSKRSIDVTNTDP- 409

PFL0020w_D2 Q**Y**LM**LYY**E**A**NT--TARY**G**IHAY-AYAVGEKDKP**VV**A**F**LQK**L**QEAN--KSSASKRSKRSTDGTTTDTL 415

PF07_0050_D2 **PY**IT**LYE**Q**A**RT---VRD**G**T-----VIGDVSDQQ**V**IA**F**FKE**L**HQQNGGKKS-VDT------------- 397

PFL1950w_D2 Q**Y**AL**LYS**YVGK--SGTI**G**LGGY------PDYKQ**VV**H**F**FEE**L**QKEYENATRSSSTTKV-------SST 403

PFF0845c_D2 **IY**QM**LY**LQ**A**QT-TAR**N**A**G**DTAF----DNPNDQY**VI**D**F**FKK**L**QKANGDNNFGVNTS------------ 399

**Loop 4**

*****  ***** : :: *****: *****

PF11_0521_D2 DAARNRVKRADGSSATRVTATTTITP**Y**ST**A**A**GYIHQE**AH--IGD**C**QK**Q**TQ**FC**KNKNGSDVS 464

var16 PTLTS--------------------P**Y**FT**A**A**GYIHQE**AR--VGE**C**EE**Q**KH**FC**TSGDKE--- 452

var13 DTTS---------------------P**Y**ST**A**A**GYIHQE**GH--VDE**C**TE**Q**TQ**FC**KNRNGNTAS 447

Var14 TDPTT--------------------P**Y**ST**A**A**GYIHQE**I--GNAG**C**QI**Q**KH**FC**DD------- 422

Var31 GAPSG----------------NSNTV**Y**ST**A**A**GYIHQE**AH--IDD**C**NK**Q**NV**FC**EKKKGGN-- 467

var27 PIT----------------------P**Y**ST**A**A**GYIHQE**I--GYGG**C**QE**Q**TQ**FC**EKKHGATST 458

Var1 PTVTS--------------------P**Y**ST**A**A**GYIHQE**V-PHM-Q**C**NT**L**TE**FC**DN------- 436

var12 ASVAK------------------NDV**Y**ST**A**A**GYVHQE**MGPHM-E**C**KT**Q**TE**FC**EKTDEQY-- 455

var17 -------------------------P**Y**ATPA**GYIHQE**AR--VGK**C**LE**Q**NV**FC**DN-NG---- 431

var44 PNTDV---------------------**Y**ST**A**A**GYIHQE**AR--VGK**C**LE**Q**NV**FC**NT-NG---- 436

Var2 -------------------------A**Y**ST**A**E**GY**V**HQE**L-PNMG-**C**NV**Q**TQ**FC**KHKNGSTSS 449

Var7 TDDTT------------------PTV**Y**ST**A**A**GYIHQE**M-PIVG-**C**KG**Q**EV**FC**DNNGNK--- 468

var8 -------------------------I**Y**ST**A**E**GY**V**HQE**L-PNMG-**C**KE**Q**IR**FC**EKKNGSTSS 421

var11 ATKS---------------------P**Y**AT**A**D**RYIHQE**I-GNVG-**C**NI**Q**NE**FC**FKKNGSG-- 446

Var19 AAPIT--------------------P**Y**ST**A**A**GYIHQE**LGKTVG-**C**NI**Q**TK**FC**KHKIGSKAS 461

var15 TSKSD--------------------V**Y**ETTA**GYIHQE**ARTR--E**C**LG**Q**NV**FC**NNNG---NN 436

Var20 TTPNT--------------------L**Y**SS**A**A**GYIH**H**E**LGRTVG-**C**NT**Q**KE**FC**YSKNG---- 455

var6 TAPNT--------------------P**Y**AT**A**A**GYIHQE**LQ-NM-E**C**QV**Q**KE**FC**EKKRG---- 461

var10 SGKTT---------------------**Y**NT**A**A**GYIHQE**AR--VGE**C**EV**Q**KY**FC**N-TNG---- 427

PF08_0141_D2 -------------------------V**Y**ST**A**A**GY**V**HQE**A--TMN-**C**EK**Q**TQ**FC**KNKNGVKAA 460

PF11_0008_D4 -------------------------I**Y**TT**A**A**G**F**IHQE**AH--MTD**C**QK**Q**TI**FC**KN------T 434

PF11_0521_D3 -------------------------I**Y**ST**A**A**GYIHQE**AH--IGD**C**KE**Q**HV**FC**DKKNGDKPT 414

PF13_0003_D2 KGNKT---------------------**Y**DT**A**E**GY**V**HQE**AH--ISD**C**QK**Q**TQ**FC**KKRNGEIPS 427

PF13_0003_D5 -------------------------I**Y**YT**A**A**GYIHQ**QAK--YLD**C**TQ**Q**TH**FC**DKKNGETLP 441

PFD0020c_D2 MTPNT--------------------L**Y**SS**A**A**GYIHQE**LGKTVG-**C**NT**Q**KE**FC**DNKKG---- 430

PFD1235w_D3 TGVHT--------------------V**Y**ST**A**A**GYIHQE**ARTR--E**C**QE**Q**RE**FC**DKKNGIDNT 436

PFD1235w_D2 DATDD----------------TTPTV**Y**ST**A**A**GY**V**HQE**AH--IGD**C**KE**Q**HV**FC**DN-NGN--- 465

PF08_0140_D2 LTPIT--------------------L**Y**SS**A**A**GYIHQE**LGKTVG-**C**NT**Q**KE**FC**KNGSG---- 435

PFF0010w_D2 MTPNT--------------------P**Y**ST**A**A**GYIH**H**E**LGKTVG-**C**NV**Q**TK**FC**KHKIGSKAS 472

PFF1580c_D2 TLTS---------------------P**Y**SS**A**A**GYIHQE**I-GNVG-**C**NV**Q**TQ**FC**EKKNGVIPT 447

PFL0020w_D2 TPTT---------------------P**Y**ST**A**E**GY**V**HQE**A--TMN-**C**DT**Q**TQ**FC**EKKHGGTTP 452

PF07_0050_D2 -------------------------V**Y**ST**A**A**GY**V**HQE**A--IMN-**C**EK**Q**TQ**FC**KNKNGENSI 430

PFL1950w_D2 ASPIT--------------------P**Y**SSPE**GYIHQE**L--PITG**C**QK**Q**KE**FC**YYKNGLTSR 442

PFF0845c_D2 -------------------------P**Y**FTPA**GYIHQE**AR--VGE**C**EV**Q**KH**FC**NNNGNQ--- 430

***** :**.**  **::*::** ***** ******

PF11_0521_D2 D--TEADP-T**Y**A**F**RDK**P**HD**H**DT**AC**K**C**KDRQPEL-----VTE--KKKDDEGEEQ------- 507

var16 -----NKD-K**Y**A**F**REK**P**KD**H**DD**AC**A**C**ENN------------------------------- 475

var13 G---KEDD-N**Y**T**F**KDP**P**PK**Y**AN**AC**K**C**DDR------------------------------- 472

Var14 -----NKD-K**Y**V**F**REK**P**KD**H**DE**AC**N**C**T--------------------------------- 443

Var31 ----DNNE-K**Y**A**F**HPE**P**YD**H**KK**AC**A**C**DGRNPDVKVLEDPCKMVQKLISEQIEK------- 515

var27 S-TTKENK-E**Y**T**F**KQP**P**PE**Y**AT**AC**D**C**INR------------------------------- 485

Var1 -----KNG-K**Y**T**F**EDP**P**TL**Y**KD**AC**N**C**E--------------------------------- 457

var12 ------NE-N**Y**T**F**KNP**P**PQ**Y**KD**AC**I**C**--N------------------------------- 475

var17 -----NNE-K**Y**A**F**KKT**P**PL**Y**KY**AC**E**C**KPK------------------------------- 454

var44 -----NNE-K**Y**A**F**KNP**P**PQ**Y**KD**AC**E**C**KPP------------------------------- 459

Var2 DGKDNDKE--**Y**A**F**REK**P**HD**H**DD**KC**T**C**T--------------------------------- 474

Var7 -------E-K**Y**A**F**KNP**P**NV**Y**DE**AC**K**C**ENN------------------------------- 489

var8 -GKDSDKE--**Y**A**F**REK**P**HD**H**EKE**C**N**C**Q--------------------------------- 445

var11 G-KVNNNE-K**Y**A**F**KYP**P**PD**Y**VE**AC**K**C**MKR------------------------------- 473

Var19 G---TENK-E**Y**A**F**RDK**P**HD**H**DE**AC**A**C**RPP------------------------------- 486

var15 E---------**Y**A**F**SLT**P**HE**Y**KH**AC**K**C**NEN------------------------------- 456

Var20 ---------K**Y**A**F**KDP**P**KG**Y**EE**AC**K**C**NDR------------------------------- 475

var6 -----DKK-K**Y**A**F**RHQ**P**YD**Y**GD**A**LR**C**DSR------------------------------- 484

var10 -----NQD-K**Y**V**F**REK**P**KD**H**DE**AC**K**C**KDR------------------------------- 450

PF08_0141_D2 NDAEDVN---**Y**T**F**KDT**P**NR**Y**DVV**C**K**C**KDRP-------------------------EQQIK 492

PF11_0008_D4 SYNDKKK---**Y**A**F**RHP**P**HD**H**DD**AC**A**C**RPPSTPVDVSRKLDTQRDPKKEESEPESEEEEDD 491

PF11_0521_D3 NGEEKVDNEK**Y**A**F**KHP**P**HE**Y**EV**AC**K**C**ETN------------------------------K 444

PF13_0003_D2 S-DTETDN-N**Y**A**F**RPQ**P**HD**H**DEV**C**E**C**NTR------------------------------- 454

PF13_0003_D5 SGRDNDK---**Y**A**F**KKP**P**KK**Y**ER**AC**K**C**H--------------------------------- 465

PFD0020c_D2 ---------K**Y**A**F**KHP**P**KE**Y**EE**AC**I**C**DTR------------------------------- 450

PFD1235w_D3 S---------**Y**A**F**KDP**P**HG**Y**AT**AC**D**C**INR------------------------------- 456

PFD1235w_D2 ------KE-K**Y**A**F**KNP**P**NV**Y**VE**AC**K**C**MTR------------------------------- 487

PF08_0140_D2 -------E-K**Y**A**F**KHP**P**KE**Y**KD**AC**S**C**NTR------------------------------- 456

PFF0010w_D2 G---TENK-E**Y**A**F**REK**P**YD**H**DD**AC**A**C**RPP------------------------------- 497

PFF1580c_D2 TGSGTNNK-N**Y**A**F**KNT**P**KD**H**DE**AC**E**C**ESR------------------------------- 475

PFL0020w_D2 TGTNDTDA-P**Y**T**F**KQP**P**PE**Y**KD**AC**E**C**DGK------------------------------- 480

PF07_0050_D2 SGTQNNQ--K**Y**A**F**MQP**P**KG**Y**EK**AC**S**C**EDY------------------------------- 457

PFL1950w_D2 SSDAKENK-N**Y**A**F**KNP**P**HG**Y**DL**AC**T**C**NTR------------------------------- 470

PFF0845c_D2 -------D-K**Y**S**F**RNQ**P**YD**H**EE**AC**A**C**KKN------------------------------- 451

*** *** ***** **:** **::** *****

PF11_0521_D2 EDEPPKPKPPSTPNP-------------- 522

var16 -TKPQPAPKKEEE---------------- 487

var13 -KPEAPPKPAQK----------------- 483

Var14 --ENVEKPKKEE----------------- 453

Var31 -NNIHN----------------------- 520

var27 -SQTEEPKKKEE----------------- 496

Var1 -SRPQVPPKKKED---------------- 469

var12 -TRPPPKEDSRKRSE-------------- 489

var17 -AQP------------------------- 457

var44 -QQENP----------------------- 464

Var2 -DKSTP----------------------- 479

Var7 -TKPPP----------------------- 494

var8 -SRNREPPPRR------------------ 455

var11 -EAP------------------------- 476

Var19 -STP------------------------- 489

var15 -KASSP----------------------- 461

Var20 -NPKPQPAPKKEDE--------------- 488

var6 -TAPEPKKVEKKE---------------- 496

var10 -PQ-------------------------- 452

PF08_0141_D2 KKEVEDACKIAQDI--------------- 506

PF11_0008_D4 AEEEEEPA--------------------- 499

PF11_0521_D3 KPEAPPPP--------------------- 452

PF13_0003_D2 -QKTKVRK--------------------- 461

PF13_0003_D5 --EKQEP---------------------- 470

PFD0020c_D2 -QKAQKP---------------------- 456

PFD1235w_D3 -SQTEEP---------------------- 462

PFD1235w_D2 -EAPPPPTTPSTPNP-------------- 501

PF08_0140_D2 -DKKSEAPVTKKEEA-------------- 470

PFF0010w_D2 -KPTGG----------------------- 502

PFF1580c_D2 -PQVPP----------------------- 480

PFL0020w_D2 -SPQAPKKEEEKKDA-------------- 494

PF07_0050_D2 -YKAADP---------------------- 463

PFL1950w_D2 -DQQTDGRGRVAVNPDDNIITPGRIDNGE 498

PFF0845c_D2 -TKAPEKKKEETPPAGP------------ 467
