# Supplementary figures and images for: High Throughput Functional Assays of the Variant Antigen PfEMP1 Reveal a Single Domain in the 3D7 Plasmodium falciparum Genome that Binds ICAM1 with High Affinity and Is Targeted by Naturally Acquired Neutralizing Antibodies
Source: PLoS Pathog. 2009 Apr 17;5(4):e1000386. doi: 10.1371/journal.ppat.1000386 (PMC2663049; doi:10.1371/journal.ppat.1000386)

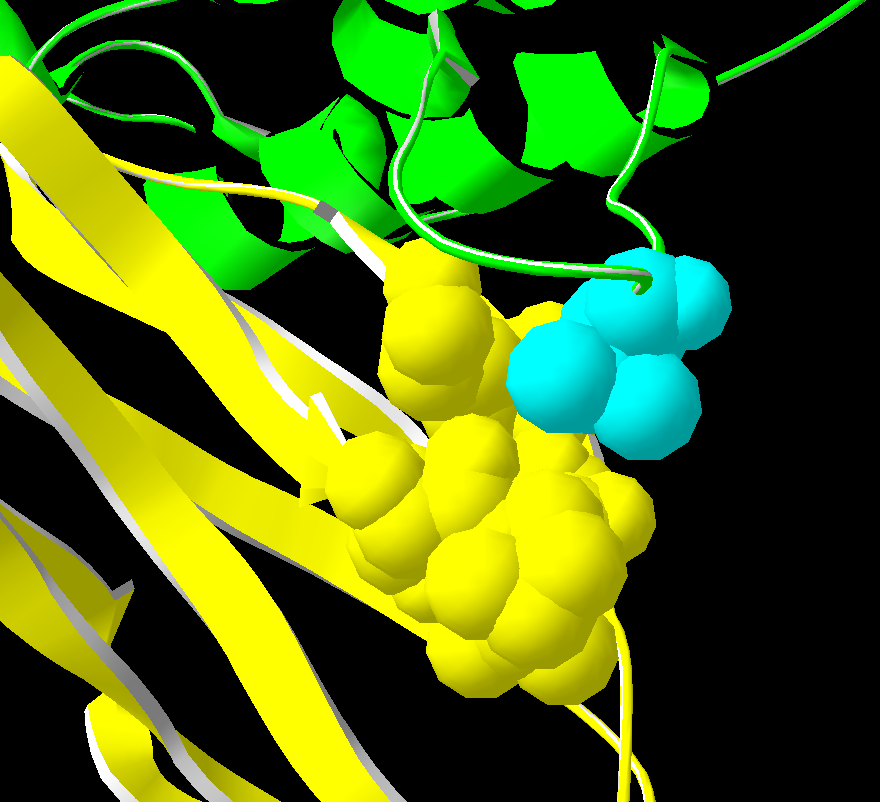

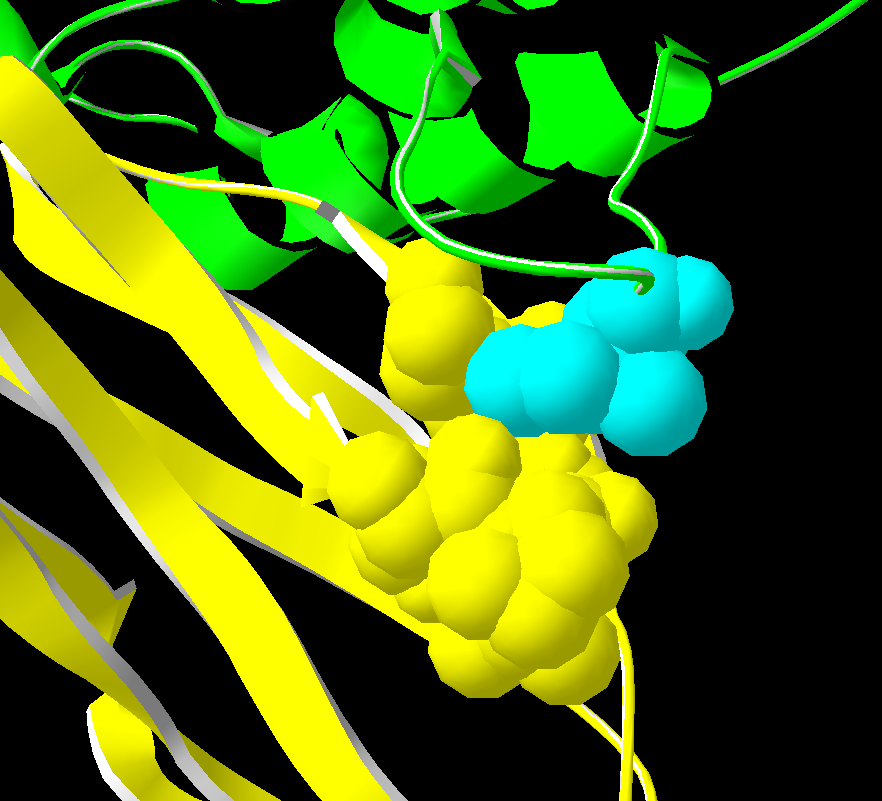


**Ile**

**Val**

**A**

**B**

Supplement: Figure S3 — Model of Ile and Val amino acid residues in “loop 3” of DBLβC2 domain interacting with ICAM1. Illustration of interaction and amino acid residue substitution were created using DBLβC2::ICAM1 complex modeled in [32] and Deep View/Swiss-pdb viewer program (v.3.7). Green - DBLβC2 domain, yellow - ICAM1 molecule. ICAM1 residues within 5 angstrom distance from Ile/Val residue of DBLβC2 domain shown in spacefill shape. (0.31 MB DOC) [file ppat.1000386.s003.doc]
