# Supplementary material for: High Throughput Functional Assays of the Variant Antigen PfEMP1 Reveal a Single Domain in the 3D7 Plasmodium falciparum Genome that Binds ICAM1 with High Affinity and Is Targeted by Naturally Acquired Neutralizing Antibodies
Source: PLoS Pathog. 2009 Apr 17;5(4):e1000386. doi: 10.1371/journal.ppat.1000386 (PMC2663049; doi:10.1371/journal.ppat.1000386)
Supplement: Figure S4 — Alignment of two DBLβC2 domain sequences (bottom) and three DBLβ domain sequences (previously described as lacking adjacent C2 domains) with surrounding sequences (top). Color font: Orange - cysteines not present in any sequence in Figure S2, which may be involved in disulfide bonding. Other letter colors are as in Figure S2. Gray highlights indicate extra N-terminal and C2 domain sequences. Yellow highlight within C2 domain indicate “loop 4” sequence. The results demonstrate that all sequences share C2 and additional N-terminal sequence (discussed in the text) with multiple conserved residues. Green highlight above the sequence alignment indicates alpha-helix predicted for all (n = 36) DBLβC2 domains described in this work [46]. (0.06 MB DOC) [file ppat.1000386.s004.doc]

**CLUSTAL 2.0.10 multiple sequence alignment of DBLβC2 domains**

----alpha helix-----

MC_DBL3 --------------KF**C**PPPPP-PMTC**V**EK**IA**KELRVE**A**EGK**I**NN--------------E 31

PFE1640w_DBL6 CE**C**PEISKNESKTHTKKIPAPKIPMNC**V**EKA**A**YYLSKE**A**ENN**I**ER--------------T 46

A4_DBL5 --------------CK**C**AIPSE-PMSC**V**E**QIA**KHLREK**A**EKN**V**KIY-----------ESS 34

11_0521_DBL2C2 --------------**NPC**AKPHGKKLAT**V**K**QIA**QYYKRK**A**YIQ**L**NE---------RGSRSA 37

var31_DBL2C2 --------------**NPC**GKTDGTTVR-AK**QIA**KKFQRD**A**KTQ**M**KNNTRNDGTGRKGAHNS 45

***** : :::.* .***** :::

MC_DBL3 **LK**GNGKD---**F**NGKCNNVKKKNGA**V**IGEES**C**KFEQTYE**N**-SVNNINNK**C**KDNQNE----RFKI 86

PFE1640w_DBL6 **LK**QK**I**T----**H**SNC---VKETDNS**F**SSNNR**C**DPNKPYAPDKYIGRRNP**C**GN**R**EQN----RFKL 98

A4_DBL5 **LK**GTPAK---SKNDC---TKIDEA**I**KGDNGSK**I**INK-**S**ILDSTFASN**C**EQSE**K**DATD--RLKI 88

11_0521_DBL2C2 **LK**GD**A**SQ-GQ**Y**DR-----GGKADD**F**K-TK**LC**E**I**NEK**HSN**-ARSNSLNP**C**NG**K**DNNK--VRFNV 90

var31_DBL2C2 **LV**GD**I**SK-AY**F**KN-----GGQGSD**L**KGDK**IC**D**I**NTS**HSN**DSRGNGGGP**C**IG**K**DGNQGGDRMKI 102

*****  : : .. . .. *:::

MC_DBL3 GQKWN-FKYIGTIR--KDLCIPPRREHMCLDDLSMLGRTTISDS---------SALLKKI 134

PFE1640w_DBL6 DSEWKCYKNIKLYQEKKRVCIPPRRENMCISNLDEIEIAKVNQS---------NYLLNMV 149

A4_DBL5 GKQWQFNKINGTET---KLYVPPRRKDMCFNDLKNIQFNEVQDS---------NSLLEKI 136

11_0521_DBL2C2 GTPWQ-SGEKIATAT--DVYLPPRRQHFCTSNLEYLINGGHQAILNVKNGKINHSFLGDV 147

var31_DBL2C2 GTPWSKVGEDKTTYS--DVYLPPRRQHMCTSNLEFLETKDTPLDGKFGVDKINHSFLGDV 160

. *. : :****:.:* .:*. : :* :

MC_DBL3 QEAAKSERDDIIRKLLEQNS-------CDEHRICDAMKYSFADLGDIIRGRDLWNKNSKQ 187

PFE1640w_DBL6 RIAARNEGIDIIKNFNSQNG-------CAMNPICDTMKYSFADLGDIVRGTDMLRIGGYL 202

A4_DBL5 QHVAKNEGIDILKKLNPQDQ-------NAFSEICDAMKYSFADLGDIIRGRSKIDPTN-- 187

11_0521_DBL2C2 LLAAKYQAQHTMKDYKSKND---------KEGICRAIRYSFADIGDIIKGTDLWDKDGGE 198

var31_DBL2C2 LLVANFEAKNIKELYKNNNDRKDLNDANDKETVCRAMKYSFADIGDIIRGTDMWDKDEGS 220

.*. : . . :: :* :::*****:***::* .

MC_DBL3 KGLQKRLEYAFINIYNKLQNDKNKYEKD-RPKYLQLRSDWWDANRKHIWNAMTCNAPDDA 246

PFE1640w_DBL6 PPVEIKLYKAFEYIYGKWRN-KNKGRNK-YNDVQTFRSAWWDANRKDIWKAMTCKAPEDA 260

A4_DBL5 ---NNKIEKELQKIFKQIQD--DNASLS-KMELPELREKWWDANRKEVWNAMTCVAPNDA 241

11_0521_DBL2C2 IKTQNHLVTIFDKIKAQLPKDIKGKYTG--TKHLELRKDWWEANRDQVWKAMQCGNDNPC 256

var31_DBL2C2 KKMDVILKKIFGKIKQELPKEIQKKYKNPDGKHTQLRKDWWEANRHQVWRAMKCAIQDGS 280

: : : * : . . . :*. **:***..:*.** * : .

MC_DBL3 KFLKKNPNDTSGSSSSKGIMTTHSNCGYDKEPPDYDYIPQPFRWMQEWSESFCKLLN--- 303

PFE1640w_DBL6 KLFRKGRMDGFES-----ITLIQDKCGHKDDPPVDDYIPQRFRWMTEWSEYYCKALKVEL 315

A4_DBL5 HLKKKKNNPGNKSQIIASQTEQTKKCSHDSEPPDYDYIPERYRFLQEWSEYYCKALK--- 298

11_0521_DBL2C2 -------------------------SGESDHTPLHDYIPQRLRWMTEWAEWYCKEQS--- 288

var31_DBL2C2 -------------------------IEKCNGIPLDDYIPQRLRWMTEWAEWFCKMQK--- 312

. * ****: *:: **:* :** .

MC_DBL3 EEMEQFEKTCGECKKNSITCEDDRNGTNCENCKNQ**C**EK**Y**KKL**I**HN**W**KLGFDKYKEI**Y**NEI 363

PFE1640w_DBL6 EKLEKLEKSCDHCKT-TNKCKNDYDKNKCEKCKTR**C**QQ**Y**DNF**I**LK**W**KTLFDIQSK**KY**KE**L** 374

A4_DBL5 EKNDEMKNDCSKCIKSGATCEKEEDKEKCKECNDK**C**KE**Y**KNIVDK**W**QSEFDQQNQL**Y**KK**L** 358

11_0521_DBL2C2 RLYDKLK-VCEECMRKGESCTKGSG--ECATCKE**AC**EE**Y**NKE**I**KK**W**EQ**QW**DAISY**KY**LM**L** 345

var31_DBL2C2 EAYNELKGKCSQCKTKDKKCTNKSD--DCNTCTE**AC**TA**Y**NRK**I**NT**W**KQ**QW**DAISD**KY**QF**L** 370

. :::: * .* .* . . .* *. *** *.. : .*: :* . * :**

MC_DBL3 **Y**NNKDSK-IN------------SNEYFKK**FL**EK**L**KDKCKE-------------------- 392

PFE1640w_DBL6 **Y**EPIDTKN-------------STYDH**V**EN**F**VQK**L**KKYKNE-------------------- 405

A4_DBL5 **Y**TQDRTHGPS----------TARRNPSIE**F**TQK**L**EDSCND-------------------- 390

11_0521_DBL2C2 **Y**AKARITAINGGPGYYNTEVQEEDKP**VV**D**FL**YN**L**YLQNGGKKGPPPDTHRVKALIARVKR 405

var31_DBL2C2 **Y**LQAKTAAANGGPHASSGDVGEKDKP**VV**N**FL**FE**L**YKQNGGKISTPSDTHPG----PRVKR 426

***** . .* :*

**Y-motif**

MC_DBL3 -------------------------LN**S**-SDKC**I**DEAT**H**---**C**TKY-K**F**S-NSE------ 413

PFE1640w_DBL6 -----------------------CSVE**S**-VSE**YLH**ETSK---**C**LNY-K**F**DENDG------ 427

A4_DBL5 -------------------------P**YS**-**A**DK**YL**DIST**H**---**C**TDY-K**F**S—ET------- 410

11_0521_DBL2C2 DAARNRVKRADGSSATRVTATTTITP**YSTAAGYIHQE**A**H**IGD**C**QKQTQ**FC**KNKNGSDVSD 465

var31_DBL2C2 GAPS-------GNSN---------TV**YSTAAGYIHQE**A**H**IDD**C**NKQNV**FC**EKKKGG---- 466

*** . :. .****. * ***

MC_DBL3 NKNHNN**YAF**KNP**P**KE**YE**K**AC**K**C**DAPDPLDN 443

PFE1640w_DBL6 SSNIRS**YAF**EET**P**KS**YK**E**AC**S**C**TLPSKNP- 456

A4_DBL5 DSRESN**YAF**SPY**P**KD**YK**E**NC**K**C**KVNTPTS- 439

11_0521_DBL2C2 TEADPT**YAF**RDK**P**HD**HD**T**AC**K**C**KDR----- 490

var31_DBL2C2 NDNNEK**YAF**HPE**P**YD**HK**K**AC**A**C**DGRNPDV- 495

. .******* ***** .**:.**  ***** *****
