# Supplementary material for: High Throughput Functional Assays of the Variant Antigen PfEMP1 Reveal a Single Domain in the 3D7 Plasmodium falciparum Genome that Binds ICAM1 with High Affinity and Is Targeted by Naturally Acquired Neutralizing Antibodies
Source: PLoS Pathog. 2009 Apr 17;5(4):e1000386. doi: 10.1371/journal.ppat.1000386 (PMC2663049; doi:10.1371/journal.ppat.1000386)
Supplement: Table S1 — Primers for PCR amplification of 3D7 DBLβC2 domains. (0.05 MB DOC) [file ppat.1000386.s005.doc]

**Gene and domain Primers 5’->3’ (Forward – top, Reverse – bottom)**

**PF11_0521-DBL2C2** cccGGATCCagAACCCGTGTGCTAAACCTCATGG

cccGAATTCcCGGATTTGGAGTAGAAGGAGG

**PF11_0521-DBL3C2** CccGGATCCagAGTGGTACTCATATCGTGAGTG

CccGAATTCcTGGAGGTGGAGGTGCTTCCGG

**PF08_0141-DBL2C2** CccGGATCCagCCCACCCCTAATCCGTGTGTCAATG

CccTCTAGAGTCCTGCGCTATTTTGCACGCATCTTC

**PF11_0008-DBL4C2** CccGGATCCag AATCCATGTGGCGACAAAAGCGCC

CccTCTAGATGCCGGCTCCTCCTCCTCTTCGGC

**PF13_0003-DBL2C2** CccGGATCCagCCACGCGCCCATAATCCGTGTG

CccGAATTCcTTTCCGTACCTTCGTCTTCTGTC

**PF13_0003-DBL5C2** CccTCCGGACCTCCTAATCCATGTGGCGACAAAG

CccTCTAGATGGTTCCTGTTTCTCGTGACATTTAC

**PFD0020c-DBL2C2** CccGGATCCagGGTAATAACGGTGGACCATGTAC

CccGAATTCcTGGTTTTTGTGCCTTCTGCCTAG

**PFD1235w-DBL2C2** CccGGATCCagAATGGTGAACCATGTACAGGTAAAG

CccGAATTCcCGGATTTGGAGTAGAAGGAGTTG

**PFD1235w-DBL3C2** CccCTTAAGCATTCCAATGCTCATAATGATTCAC

CccGAATTCcCGGCTCCTCTGTTTGCGACCTA

**PF08_0140-DBL2C2** CccGGATCCagAACCCCTGTGGAAAAAACAATAATGG

CccGAATTCcCGCCTCTTCTTTTTTTGTTACTGGTG

**PFF0010w-DBL2C2** CccGGATCCagGAAGAAGATGAAAATTGCCCC

CccGAATTCcACCTCCAGTGGGTTTTGGTGG

**PFF1580c-DBL2-C2** CccGGATCCagAGTAATCCATGTGCTACTCCTAG

CccTCTAGACGGAGGCACTTGTGGCCTAC

**PFL0020w-DBL2C2** CccGGATCCagAATCCGTGTAGCGCCCAACCTGG

CccGAATTCcCGCATCTTTTTTTTCTTCTTCCTTC

**PF07_0050-DBL2C2** CccGGATCCagAATAATCCGTGTGCCAAACCTAGTGG

CccGAATTCcTGGATCTGCCGCTTTATAATAGTCCT

**PFL1950w-DBL2C2** CccGGATCCagGAAGCGGTGGCGAACACGGTGG

CccTCTAGATTCACCGTTGTCAATGCGTCCAGGTG

**PFF0845c-DBL2C2** CccGGATCCagAACCCGTGTGCTGTAGGGAAAAAACTC

CccGAATTCcTGGTCCCGCTGGTGGTGTTTCTTCTTTC

Restriction sites are underlined. Red font indicates nucleotide substitution to eliminate NcoI site from the cloned fragment.
