# Supplementary material for: High Throughput Functional Assays of the Variant Antigen PfEMP1 Reveal a Single Domain in the 3D7 Plasmodium falciparum Genome that Binds ICAM1 with High Affinity and Is Targeted by Naturally Acquired Neutralizing Antibodies
Source: PLoS Pathog. 2009 Apr 17;5(4):e1000386. doi: 10.1371/journal.ppat.1000386 (PMC2663049; doi:10.1371/journal.ppat.1000386)
Supplement: Table S2 — Forward primers for PCR amplification of five 3D7 DBLβC2 domains in the same boundaries as active DBL2βC2PF11_0521 domain. (0.03 MB DOC) [file ppat.1000386.s006.doc]

**Supplementary Table S2. Forward primers for PCR amplification of five 3D7 DBLβC2 domains in the same boundaries as active PF11_0521-DBL2C2 domain**

**Gene and domain Primers 5’->3’**

**PF11_0521-DBL3C2** CCCGGATCCAGAATCCGTGTGTTCGTAAAGACCAG

**PFD0020c-DBL2C2** CCCGGATCCAGAACCCCTGTGGAACAAACAATAATGG

**PFD1235w-DBL2C2** CCCGGATCCAGAACCCCTGTGCTAAACCTCCTGGTAG

**PFD1235w-DBL3C2** CCCTCCGGAAATCCGTGTGCTGAAACTGGTGGTG

**PFL1950w-DBL2C2** CCCGGATCCAGAATCCATGTAGTGGCGACACAAG

Restriction sites are underlined. The same reverse primers were used for PCR as described in Table S1
