# Supplementary material for: High Throughput Functional Assays of the Variant Antigen PfEMP1 Reveal a Single Domain in the 3D7 Plasmodium falciparum Genome that Binds ICAM1 with High Affinity and Is Targeted by Naturally Acquired Neutralizing Antibodies
Source: PLoS Pathog. 2009 Apr 17;5(4):e1000386. doi: 10.1371/journal.ppat.1000386 (PMC2663049; doi:10.1371/journal.ppat.1000386)
Supplement: Supplementary file 6 [file ppat.1000386.s001.doc]

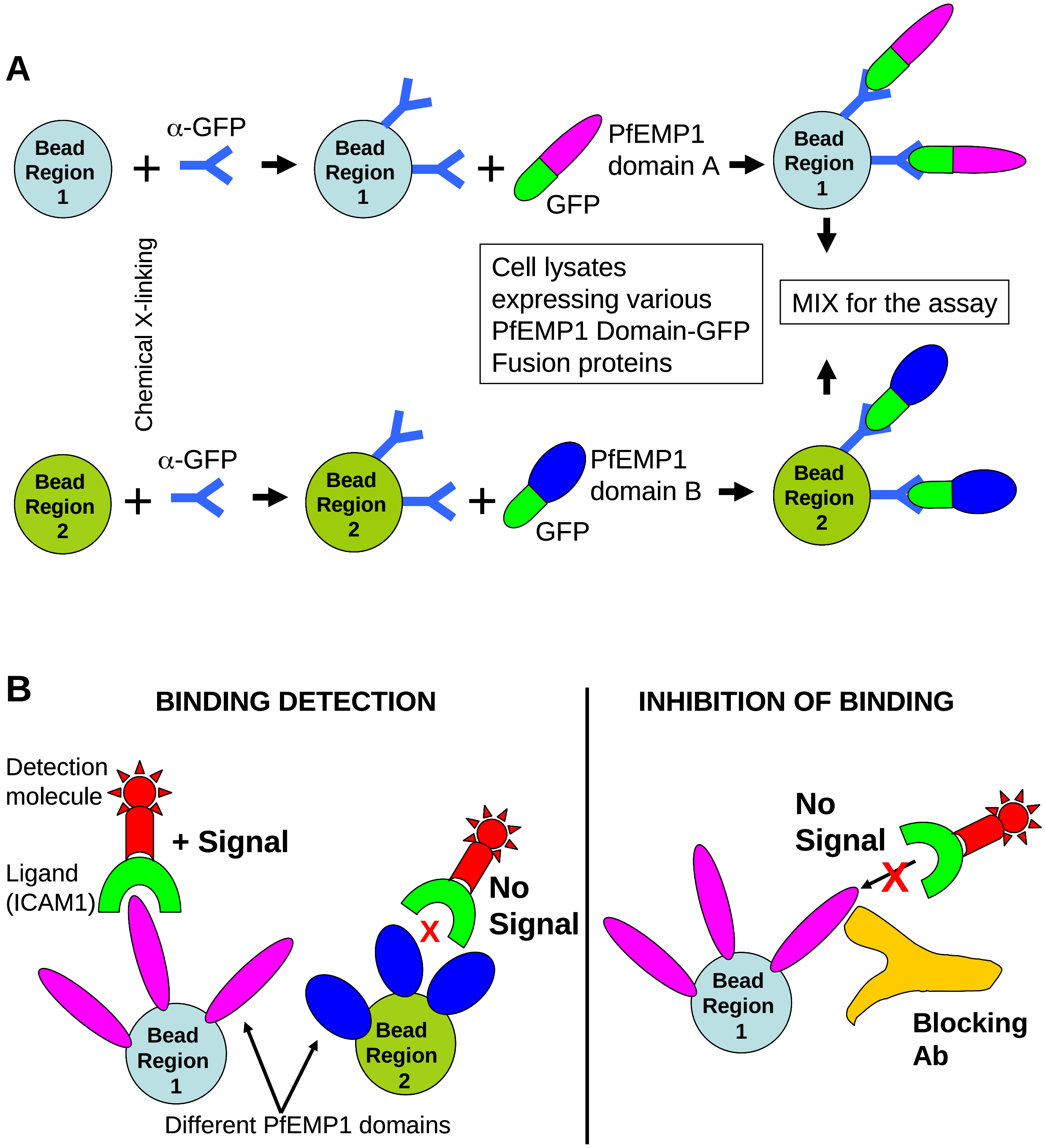
A
Bead Region 1
α-GFP
Bead Region 1
PfEMP1 domain A
GFP
Bead Region 1
Chemical X-linking
Cell lysates expressing various PfEMP1 Domain-GFP Fusion proteins
MIX for the assay
Bead Region 2
α-GFP
Bead Region 2
PfEMP1 domain B
GFP
Bead Region 2
B
BINDING DETECTION
INHIBITION OF BINDING
Detection molecule
Ligand (ICAM1)
+ Signal
No Signal
Bead Region 1
Bead Region 2
Different PfEMP1 domains
No Signal
Bead Region 1
Blocking Ab
